# Supplementary material for: FOXA2 rewires AP-1 for transcriptional reprogramming and lineage plasticity in prostate cancer
Source: Nat Commun. 2024 Jun 8;15:4914. doi: 10.1038/s41467-024-49234-9 (PMC11162502; doi:10.1038/s41467-024-49234-9)
Supplement: Supplementary file 1 — Supplementary Information [file 41467_2024_49234_MOESM1_ESM.pdf]

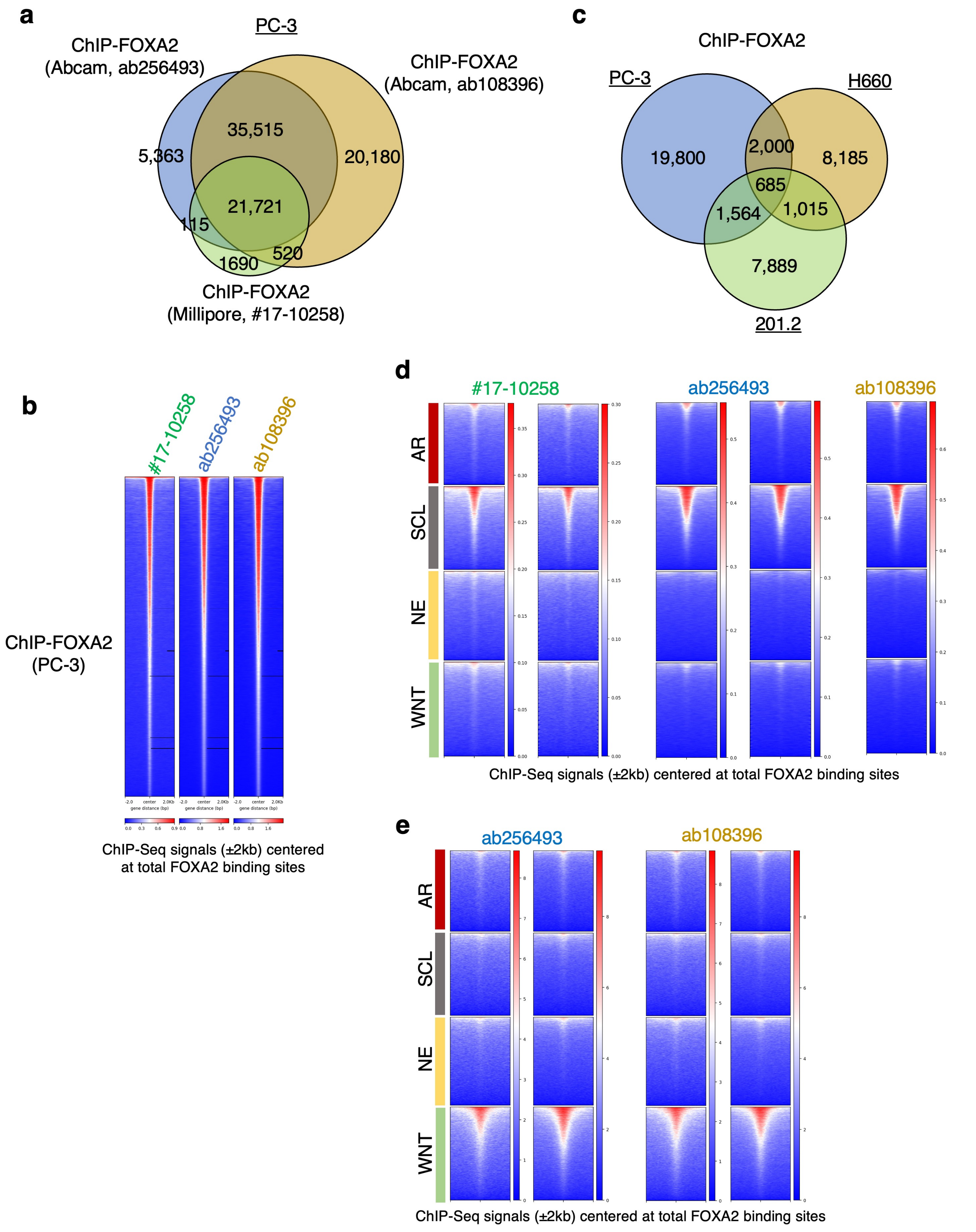
a
PC-3
ChIP-FOXA2
(Abcam, ab256493)
ChIP-FOXA2
(Abcam, ab108396)
35,515
5,363
20,180
21,721
115
520
1690
ChIP-FOXA2
(Millipore, #17-10258)
c
ChIP-FOXA2
PC-3
H660
19,800
2,000
8,185
685
1,564
1,015
7,889
201.2
b
#17-10258
ab256493
ab108396
ChIP-FOXA2
(PC-3)
ChIP-Seq signals (±2kb) centered
at total FOXA2 binding sites
d
#17-10258
ab256493
ab108396
AR
SCL
NE
WNT
ChIP-Seq signals (±2kb) centered at total FOXA2 binding sites
e
ab256493
ab108396
AR
SCL
NE
WNT
ChIP-Seq signals (±2kb) centered at total FOXA2 binding sites

**Supplementary Fig. 1| FOXA2 cistrome in PCa models**

**a**, ChIP-seq analyses of FOXA2 were conducted in PC-3 cells with indicated commercially available ChIP-grade antibodies. The Venn diagram shows the overlap of binding sites. **b**, Heatmap view for the FOXA2 binding intensity at all those sites, indicating consistent chromatin binding profiles from all three antibodies. **c,** The Venn diagram view for FOXA2 ChIP-seq peaks in PC-3, NCI-H660, and 201.2 models. **d**, **e**, Heatmap view for the FOXA2 binding intensity at lineage-specific ATAC-signature sites [1] using three different antibodies with additional replicates for PC-3 cells (d) and two different antibodies for 201.2 tumor samples (e).

**a**

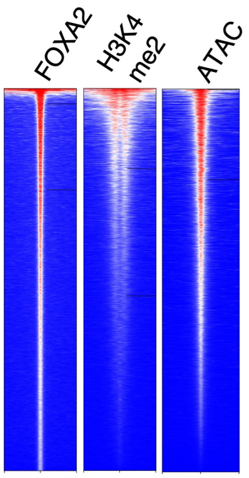


**b**

Mass-spectrometry

| Rep1/Rep2(Methyl) |
|---|
| R208me, R208me2 |
| R228me |
| K265me, K265me2 |
| K270me2 |
| K280me, K280me2 |
| K321me, K321me2 |

**Supplementary Fig. 2| FOXA2 binds to enhancers marked by high levels of H3K4me2 and ATAC signal**

**a**, Heatmap view for FOXA2, H3K4me2, and ATAC (Assay for Transposase-Accessible Chromatin using sequencing) peaks in NCI-H660 cells centered at the FOXA2 sites in NCI-H660. **b,** Mass-spectrometry analysis was performed on immunoprecipitated FLAG-FOXA2 in PC3 cells stably overexpressing FLAG-tagged FOXA2. Methylation sites on various FOXA2 amino acids were consistently detected in two biological replicates (K265 methylation highlighted in yellow).

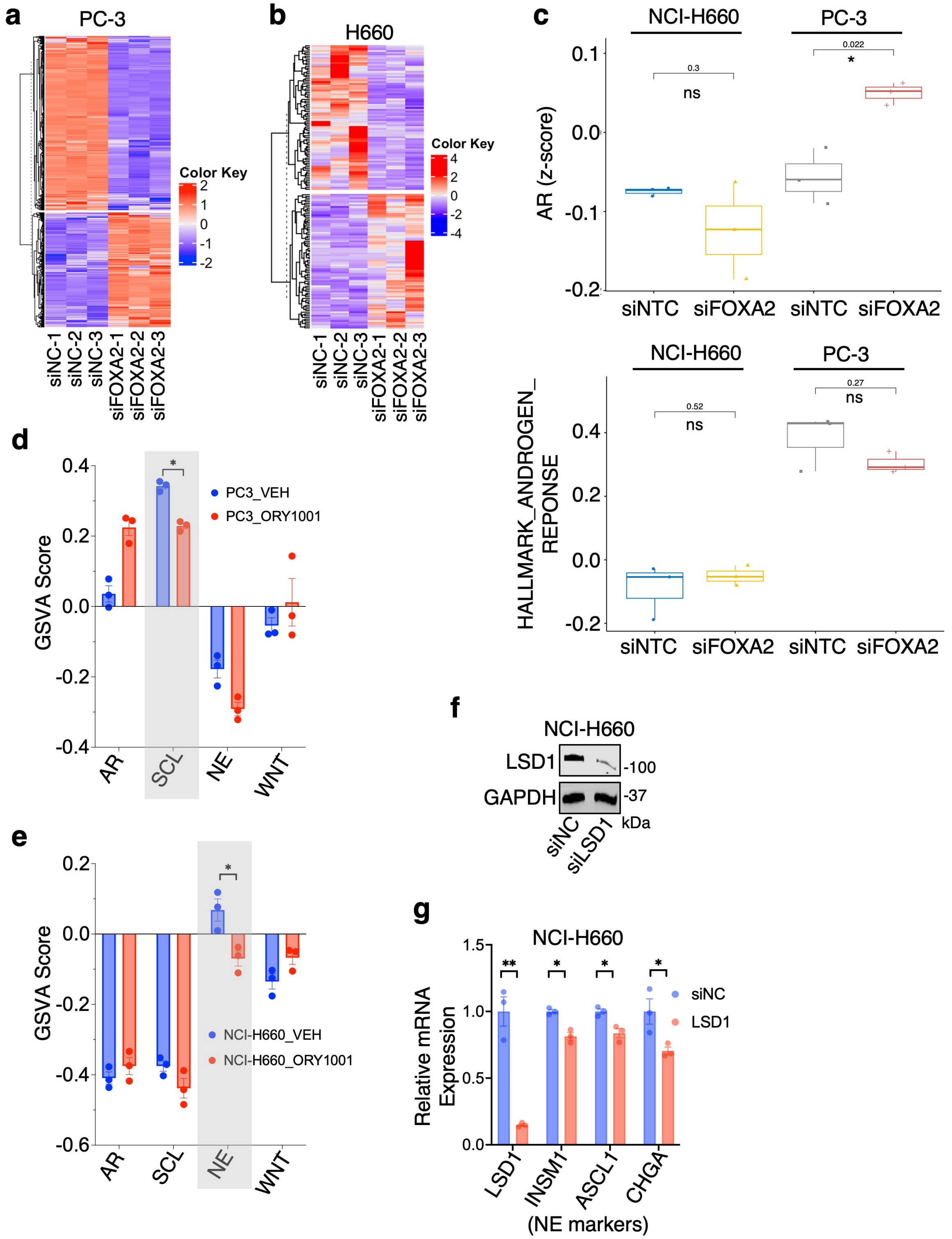


**Supplementary Fig. 3| FOXA2 transcriptome in PC-3 and NCI-H660 models**

**a**, **b**, Heatmap view for RNA-seq data showing the expression levels of FOXA2-upregulated/downregulated genes ($\log_2$(fold-change) > 1, $P < 0.05$) in PC3 cells (a) and NCI-H660 cells (b) transfected with siFOXA2 versus

siNTC. **c**, Box plot for AR expression (upper panel) and Hallmark androgen response gene signature scores (lower panel, d) in PC-3/NCI-H660 cells transfected with siFOXA2 versus siNTC (n=3 independent samples; center: median; box: $25^{th}$ to $75^{th}$ IQR; whiskers: 1.5x IQR; outliers: individual data points; statistical significance determined by unpaired two-sided *t*-test). **d**, **e**, GSVA scores for subtype-specific transcriptional signatures (based on gene annotation nearby lineage-specific ATAC signature sites [1]) in PC3 cells (d) or NCI-H660 cells (e) treated with/out LSD1 inhibitor, ORY1001 (n=3 independent samples; data represented as mean ± SEM; statistical significance determined by unpaired two-sided *t*-test). **f**, Immunoblotting for LSD1 in NCI-H660 cells transfected by siNTC or siLSD1 (n=3 independent experiments). **g**, qRT-PCR for indicated genes in these cells (n=3 independent samples; data represented as mean ± SEM; statistical significance determined by unpaired two-sided *t*-test). ns ($P>0.05$), * ($0.01<P<0.05$), ** ($0.001<P<0.01$), *** ($P<0.001$), and **** ($P<0.0001$) were used to indicate the levels of *P*-value. Source data are provided as a Source Data file.

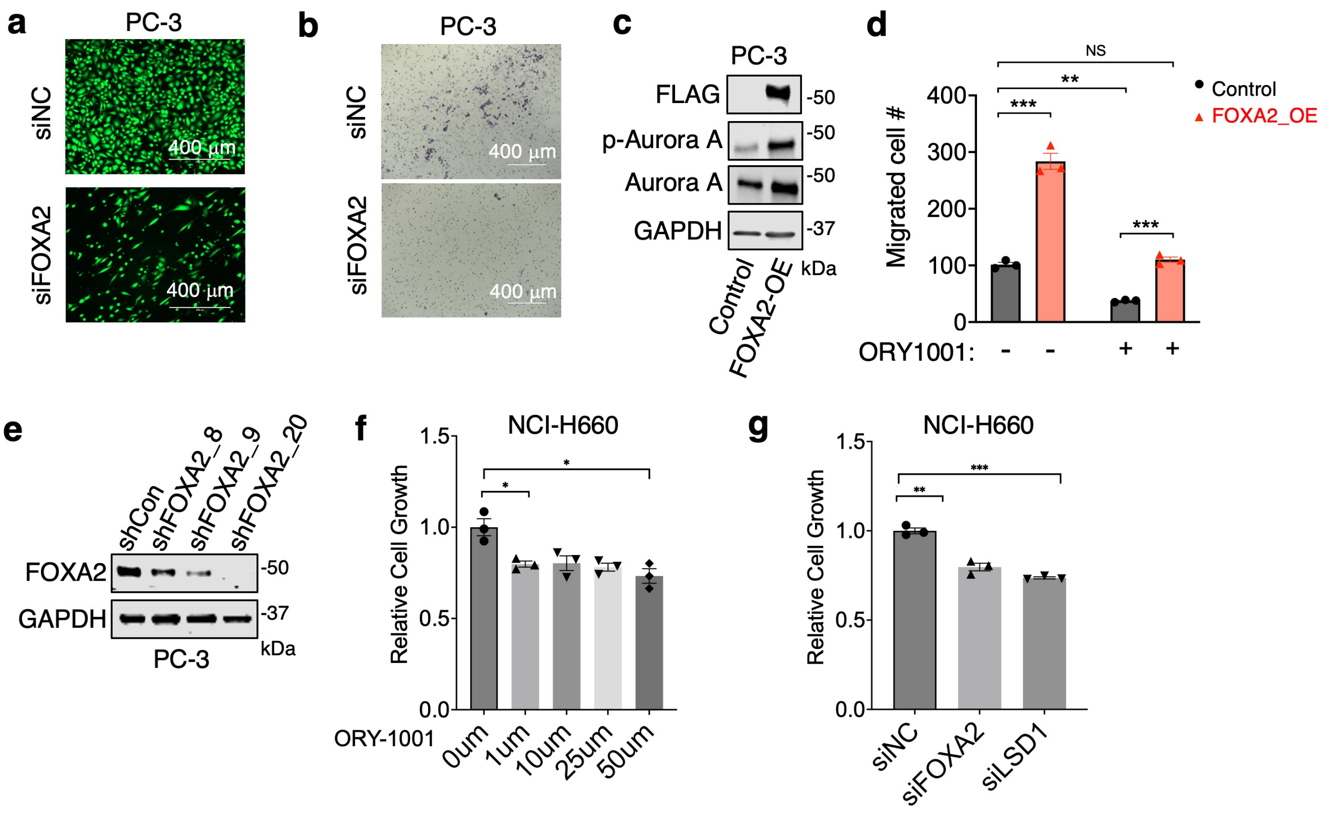


**Supplementary Fig. 4| FOXA2 promotes CRPC tumor progression**

**a**, **b**, Transwell migration assay (a) or Boyden chamber invasion assay (b) in PC-3 cells transfected with siFOXA2 versus siNTC. **c**, Immunoblotting for indicated proteins in PC-3 cells stably overexpressing FOXA2 (FOXA2-OE) versus control stable cells (n=3 independent experiments). **d**, Transwell migration assay in control PC-3 or PC-3-FOXA2-OE cells treated with /out ORY-1001 (10μM for 2d) (n=3 independent samples; data represented as mean ± SEM; statistical significance determined by unpaired two-sided *t*-test). **e**, Immunoblotting for FOXA2 in PC-3 cells stably infected with three lentiviral shRNAs against FOXA2 versus non-target-control (NTC) (n=3 independent experiments). **f**, **g**, Proliferation assay for NCI-H660 cells treated with ORY-1001 (10μM for 3d) (f) or transfected with siFOXA2 or siLSD1 (g) (n=3 independent samples; data represented as mean ± SEM; statistical significance determined by unpaired two-sided *t*-test). ns ($P$>0.05), * (0.01<$P$<0.05), ** (0.001<$P$<0.01), *** ($P$<0.001), and **** ($P$<0.0001) were used to indicate the levels of *P*-value. Source data are provided as a Source Data file.

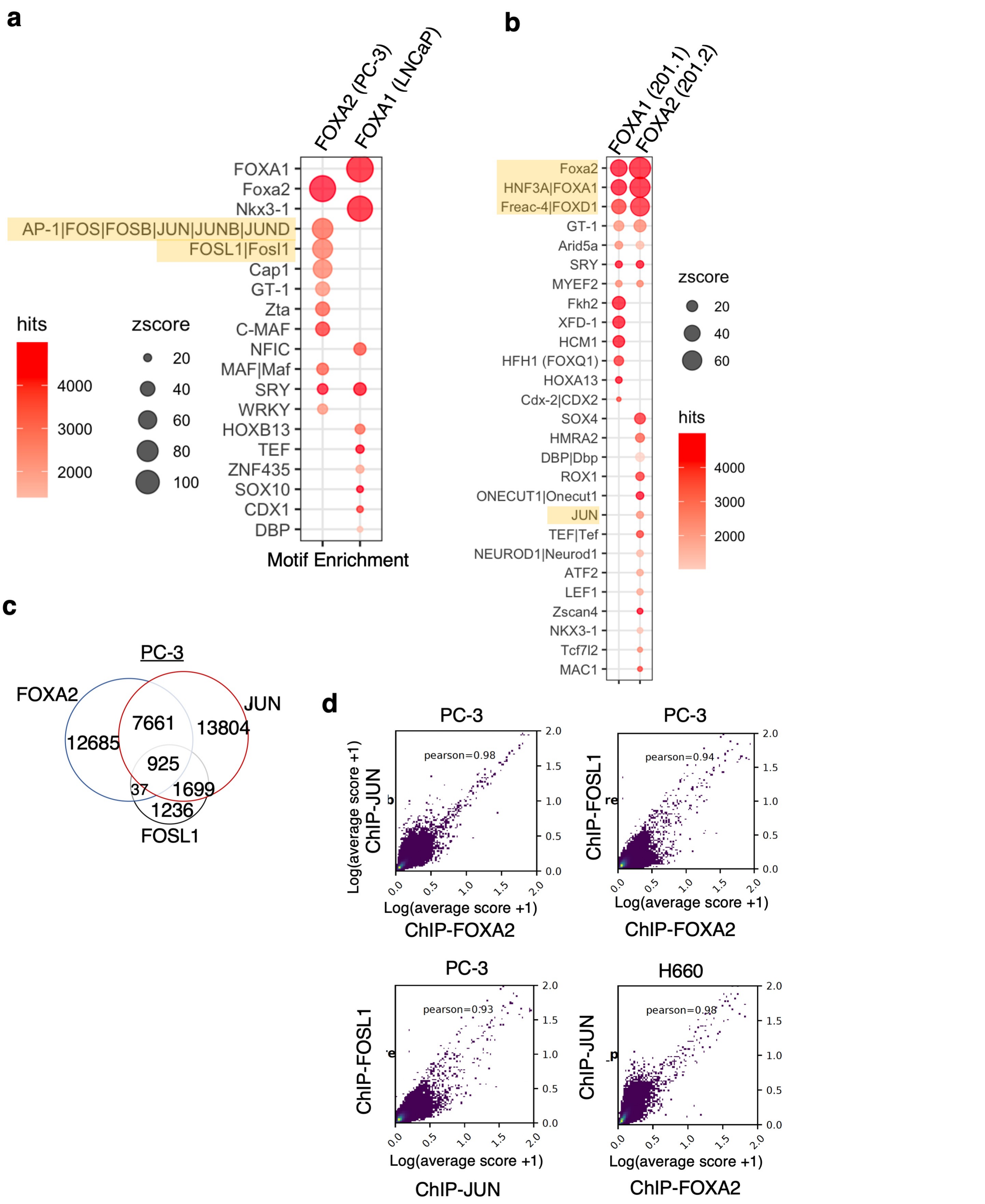


**Supplementary Fig. 5| FOXA2 and JUN chromatin binding sites are highly correlated**

**a**, **b**, Motifs enrichment analyses for ChIP-FOXA2 sites in PC-3 and ChIP-FOXA1 sites in LNCaP cells (a) or

201.1 and 201.2 tumor samples (b) by SeqPos motif tool. **c**, Venn diagram for FOXA2, JUN, or FOSL1 binding sites in PC-3 cells. **d,** A scatterplot for the correlation between two indicated ChIP-seq profiles based on read coverage within genomic regions using a bin size of 5kb in PC-3 and NCI-H660 cells. Pearson correlation coefficient was calculated. multiBigwigSummary and plotCorrelation tools were used for this analysis.

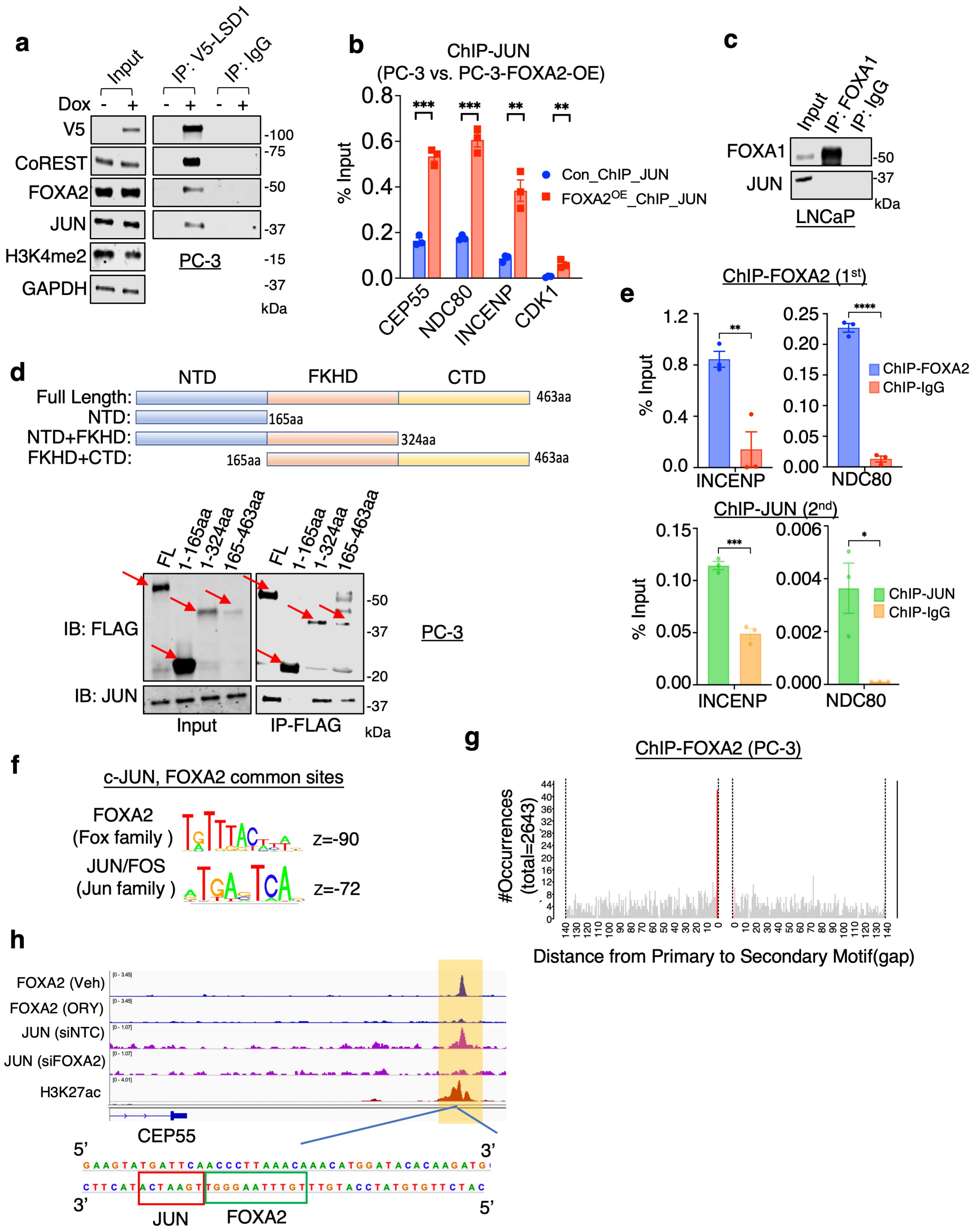
a
Input
IP: V5-LSD1
IP: IgG
Dox - + - + - +
V5
CoREST
FOXA2
JUN
H3K4me2
GAPDH
-100
-75
-50
-37
-15
-37
kDa
PC-3
b
ChIP-JUN
(PC-3 vs. PC-3-FOXA2-OE)
% Input
Con_ChIP_JUN
FOXA2OE_ChIP_JUN
CEP55
NDC80
INCENP
CDK1
c
Input
IP: FOXA1
IP: IgG
FOXA1
JUN
-50
-37
kDa
LNCaP
d
NTD
FKHD
CTD
Full Length:
NTD:
NTD+FKHD:
FKHD+CTD:
463aa
165aa
324aa
FL
1-165aa
1-324aa
165-463aa
IB: FLAG
IB: JUN
Input
IP-FLAG
PC-3
kDa
e
ChIP-FOXA2 (1st)
% Input
INCENP
NDC80
ChIP-FOXA2
ChIP-IgG
ChIP-JUN (2nd)
ChIP-JUN
ChIP-IgG
f
c-JUN, FOXA2 common sites
FOXA2
(Fox family )
z=-90
JUN/FOS
(Jun family )
z=-72
g
ChIP-FOXA2 (PC-3)
#Occurrences
(total=2643)
Distance from Primary to Secondary Motif(gap)
h
FOXA2 (Veh)
FOXA2 (ORY)
JUN (siNTC)
JUN (siFOXA2)
H3K27ac
CEP55
5'
3'
GAAGTATGATTCAACCCTTAAACAAACATGGATACACAAGATG
CTTCATACTAAGTTGGGAATTTGTTTGTACCTATGTGTTCTAC
3'
5'
JUN
FOXA2

**Supplementary Fig. 6| FOXA2 interacts with JUN on chromatin**

**a,** Immunoblotting for indicated proteins that were co-immunoprecipitated with V5 in PC-3 stable cells overexpressing doxycycline-inducible V5-tagged LSD1 (treated with 0.5ug/ml doxycycline to induce LSD1 overexpression, n=3 independent experiments) . **b,** ChIP-qPCR for validating the binding of JUN at the identified FOXA2 target sites in PC-3 cells stably overexpressing FOXA2 (n=3 independent samples; data represented as mean ± SEM; statistical significance determined by unpaired two-sided *t*-test). **c,** Immunoblotting for indicated proteins that were co-immunoprecipitated with FOXA1 in LNCaP cells (n=3 independent experiments). **d,** Immunoblotting for indicated proteins that were co-immunoprecipitated with FLAG in PC-3 cells transiently expressing FLAG-tagged truncated FOXA2 proteins (n=3 independent experiments). **e,** ChIP-qPCR to validate the co-occupancy of FOXA2 and JUN on the indicated FOXA2 target sites PC-3 cells using a re-ChIP sequential approach with FOXA2 and JUN antibodies (n=3 independent samples; data represented as mean ± SEM; statistical significance determined by unpaired two-sided *t*-test). **f-h**, Motif enrichment analysis was performed at FOXA2 and JUN co-binding sites in PC-3 cells to identify the top-ranked enriched motifs (f), followed by the analysis of the distance from the primary to the secondary motif (gap) for composition motif enrichment (g) and a genome browser view for a FOXA2/JUN co-binding site at *CEP55* gene loci (h). ns ($P>0.05$), * ($0.01<P<0.05$), ** ($0.001<P<0.01$), *** ($P<0.001$), and **** ($P<0.0001$) were used to indicate the levels of *P*-value. Source data are provided as a Source Data file.

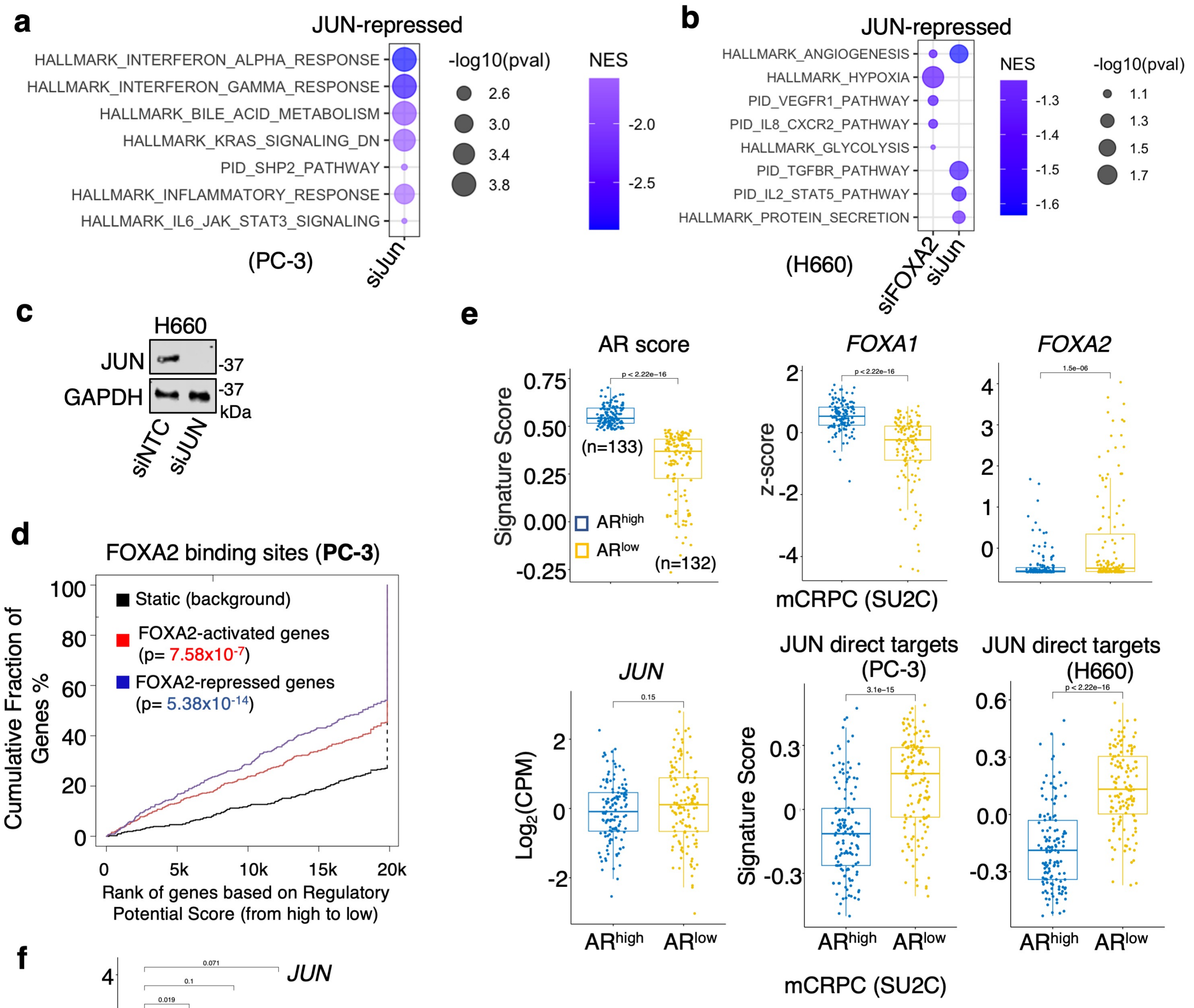


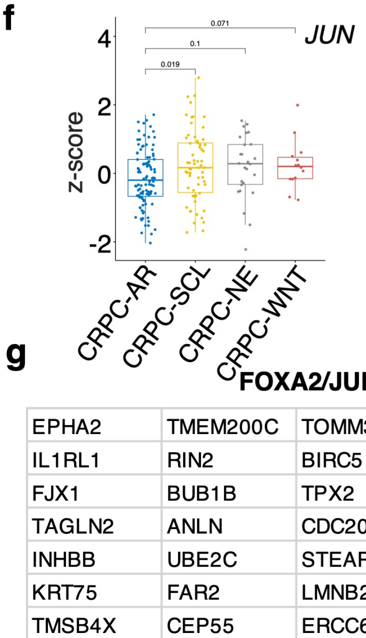


g

**FOXA2/JUN cotargets (PC-3)**

| | | | | | |
|---|---|---|---|---|---|
| EPHA2 | TMEM200C | TOMM34 | MCM7 | CENPF | IFRD2 |
| IL1RL1 | RIN2 | BIRC5 | PPARD | CDK1 | MCM2 |
| FJX1 | BUB1B | TPX2 | FOXA2 | ZWINT | BUB1 |
| TAGLN2 | ANLN | CDC20 | KIF4A | DTL | RNASEH2A |
| INHBB | UBE2C | STEAP3 | EMP2 | HJURP | NDC80 |
| KRT75 | FAR2 | LMNB2 | PLK1 | CKS1B | |
| TMSB4X | CEP55 | ERCC6L | MCAM | NUF2 | |

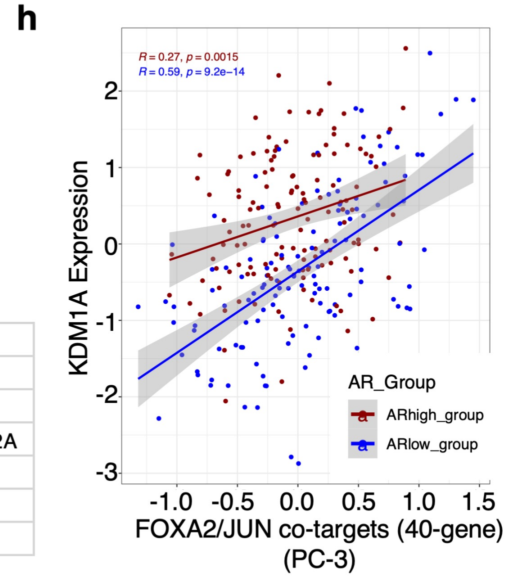

**Supplementary Fig. 7| Chromatin binding, expression, and targets of JUN in AR-low CRPC**

**a**, **b**, GSEA using HALLMARK and PID datasets to compare siJUN versus siNTC in PC-3 cells (a) or siJUN and siFOXA2 versus siNTC in NCI-H660 cells (b). **c,** Immunoblotting for JUN in NCI-H660 cells transfected with siJUN versus siNTC (n=3 independent experiments). **d,** BETA integrating ChIP-FOXA2 binding sites and RNA-seq data from PC-3 cells transfected with siNTC versus siFOXA2. **e,** Boxplots (center: median; box: 25th to 75th IQR; whiskers: 1.5x IQR; outliers: individual data points; statistical significance determined by unpaired two-sided *t*-test) for expression scores or levels of AR score, *FOXA1*, *FOXA2*, *JUN*, and JUN-direct-targets (identified from PC-3 or NCI-H660) in AR score-high (n=133) and AR-score low (n=132) CRPC patient samples (SU2C) [2]. **f,** JUN mRNA expression (z-score) in four different CRPC subtypes (CRPC-AR n=104 gens, CRPC-SCL n=62 genes, CRPC-NE n=26, and CRPC-WNT n=14) [1] using the SU2C dataset (center: median; box: 25th to 75th IQR; whiskers: 1.5x IQR; outliers: individual data points; statistical significance determined by unpaired two-sided *t*-test). **g**, **h**, FOXA2/JUN directly regulated co-targets (identified from PC3 cells, g) were correlated with *LSD1* (*KDM1A*) expression in AR score-high versus AR core-low CRPC-AR low CRPC samples (h).

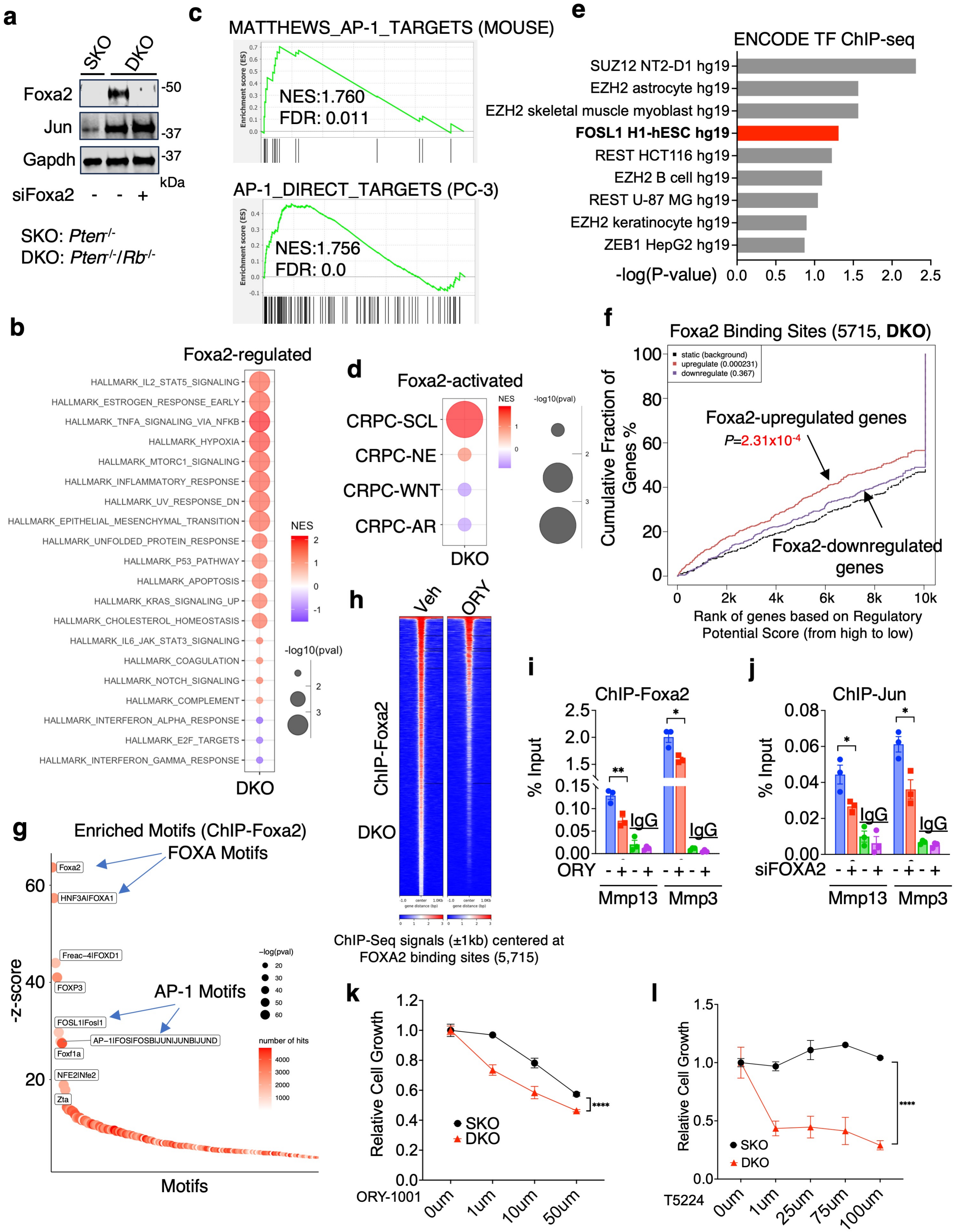

a
SKO DKO
Foxa2 -50
Jun -37
Gapdh -37
kDa
siFoxa2 - - +
SKO: Pten-/-
DKO: Pten-/-/Rb-/-
c
MATTHEWS_AP-1_TARGETS (MOUSE)
NES:1.760
FDR: 0.011
AP-1_DIRECT_TARGETS (PC-3)
NES:1.756
FDR: 0.0
e
ENCODE TF ChIP-seq
SUZ12 NT2-D1 hg19
EZH2 astrocyte hg19
EZH2 skeletal muscle myoblast hg19
FOSL1 H1-hESC hg19
REST HCT116 hg19
EZH2 B cell hg19
REST U-87 MG hg19
EZH2 keratinocyte hg19
ZEB1 HepG2 hg19
-log(P-value)
b
Foxa2-regulated
HALLMARK_IL2_STAT5_SIGNALING
HALLMARK_ESTROGEN_RESPONSE_EARLY
HALLMARK_TNFA_SIGNALING_VIA_NFKB
HALLMARK_HYPOXIA
HALLMARK_MTORC1_SIGNALING
HALLMARK_INFLAMMATORY_RESPONSE
HALLMARK_UV_RESPONSE_DN
HALLMARK_EPITHELIAL_MESENCHYMAL_TRANSITION
HALLMARK_UNFOLDED_PROTEIN_RESPONSE
HALLMARK_P53_PATHWAY
HALLMARK_APOPTOSIS
HALLMARK_KRAS_SIGNALING_UP
HALLMARK_CHOLESTEROL_HOMEOSTASIS
HALLMARK_IL6_JAK_STAT3_SIGNALING
HALLMARK_COAGULATION
HALLMARK_NOTCH_SIGNALING
HALLMARK_COMPLEMENT
HALLMARK_INTERFERON_ALPHA_RESPONSE
HALLMARK_E2F_TARGETS
HALLMARK_INTERFERON_GAMMA_RESPONSE
DKO
NES
-log10(pval)
d
Foxa2-activated
CRPC-SCL
CRPC-NE
CRPC-WNT
CRPC-AR
DKO
f
Foxa2 Binding Sites (5715, DKO)
static (background)
upregulate (0.000231)
downregulate (0.367)
Cumulative Fraction of Genes %
Foxa2-upregulated genes
P=2.31x10-4
Foxa2-downregulated genes
Rank of genes based on Regulatory Potential Score (from high to low)
g
Enriched Motifs (ChIP-Foxa2)
FOXA Motifs
Foxa2
HNF3AIFOXA1
Freac-4IFOXD1
FOXP3
AP-1 Motifs
FOSL1IFosl1
AP-1IFOSIFOSBIJUNIJUNBIJUND
Foxf1a
NFE2INfe2
Zta
-z-score
Motifs
-log(pval)
number of hits
h
Veh ORY
ChIP-Foxa2
DKO
ChIP-Seq signals (±1kb) centered at FOXA2 binding sites (5,715)
i
ChIP-Foxa2
% Input
IgG
ORY
Mmp13 Mmp3
j
ChIP-Jun
% Input
IgG
siFOXA2
Mmp13 Mmp3
k
Relative Cell Growth
SKO
DKO
ORY-1001
0um 1um 10um 50um
****
l
Relative Cell Growth
SKO
DKO
T5224
0um 1um 25um 75um 100um
****

**Supplementary Fig. 8| FOXA2 transcription activity in DKO murine PCa cell line**

**a**, Immunoblotting for Foxa2 and Jun in SKO (*Pten*$^{-/-}$) and DKO (*Pten*$^{-/-}$/*Rb*$^{-/-}$) murine PCa cells transfected with siFoxa2 versus siNTC (n=3 independent experiments). **b**, GSEA using HALLMARK and PID datasets to identify Foxa2 upregulated (siFoxa2-downregulated, red) and downregulated genes (siFoxa2-upregulated, blue). **c**, GSEA for the enrichment of Foxa2 regulated genes using MATTHEWS_AP-1_TARGETS (identified from mouse models) and our AP-1 direct targets (identified from PC-3 model) gene sets. **d,** GSEA for Foxa2-activated genes using lineage-specific signature gene sets. **e**, Prediction of potential transcription factor (TF) regulation for Foxa2-activated genes based on their nearby TF binding peaks using ENCODE TF ChIP-seq database. **f,** ChIP-seq of Foxa2 in DKO cells identified 5,715 peaks (using the antibody ab256493 from Abcam). BETA integrating ChIP-Foxa2 binding sites and RNA-seq data from DKO cells transfected with siNTC versus siFoxa2. **g**, Motif enrichment analysis of Foxa2 binding sites. **h**, ChIP-seq of Foxa2 in DKO cells treated with LSD1 inhibitor (ORY-1001, 10μM, 4h). Heatmap view for the change of Foxa2 binding intensity at 5,715 Foxa2 binding sites. **i**, ChIP-qPCR for Foxa2 binding at two Foxa2 binding sites located at Mmp3 and Mmp13 genes in DKO cells treated with/out ORY-1001 (10μM, 4h) (n=3 independent samples; data represented as mean ± SEM; statistical significance determined by unpaired two-sided *t*-test). **j** ChIP-qPCR for Jun binding at these two sites in DKO cells transfected with siNTC or siFoxa2 (n=3 independent samples; data represented as mean ± SEM; statistical significance determined by unpaired two-sided *t*-test). **k**, **l**, Viability assay for SKO and DKO cells treated with 0-50μM ORY-1001 (k) or 0-100μM T5224 (l) for 3d (n=3 independent samples; data represented as mean ± SEM; statistical significance determined by two-way ANOVA). Note: SKO (*Pten*$^{-/-}$) murine PCa cell line and DKO (*Pten*$^{-/-}$/*Rb*$^{-/-}$) murine PCa cells were cultured in DMEM supplemented with 10% FBS. ns ($P$>0.05), * (0.01<$P$<0.05), ** (0.001<$P$<0.01), *** ($P$<0.001), and **** ($P$<0.0001) were used to indicate the levels of *P*-value. Source data are provided as a Source Data file.

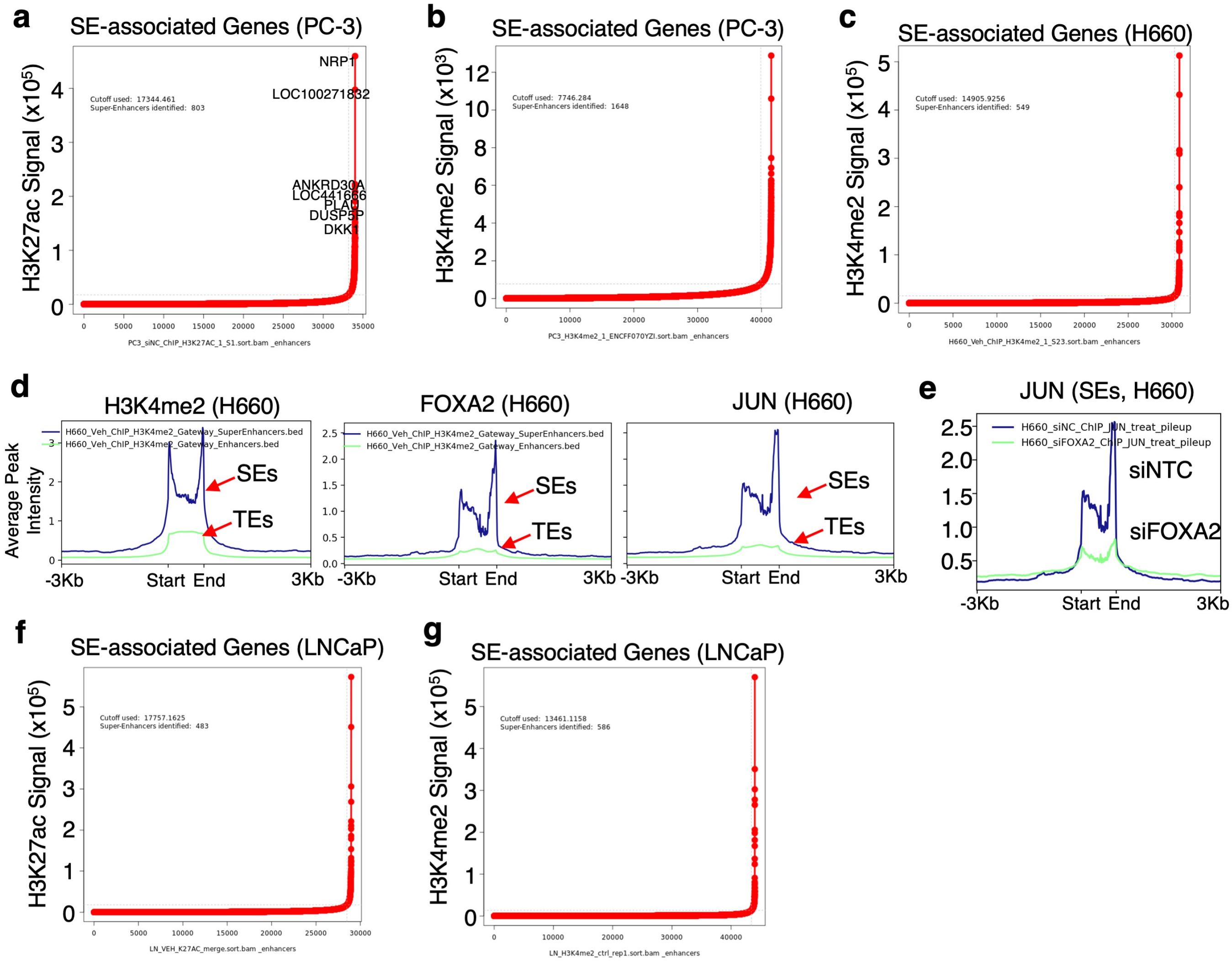


**Supplementary Fig. 9| Identification of super-enhancers in different PCa cell lines**

**a**-**c**, Model-specific super-enhancers (SEs) were identified using the ROSE algorithm based on ChIP-seq data for H3K27ac (a) and H3K4me2 (b) in PC3 cells, or H3K4me2 in NCI-H660 cells (c). **d**, Average binding intensity of indicated proteins at super-enhancers (SEs) versus typical enhancers (TEs) in NCI-H660 cells. **e**, Average JUN binding intensity at SEs in NCI-H660 cells transfected with siFOXA2 versus siNTC. **f**, **g**, Super-enhancers were identified using the ROSE algorithm based on ChIP-seq data for H3K27ac (f) and H3K4me2 (g) in LNCaP cells (GSE114268 [3]).

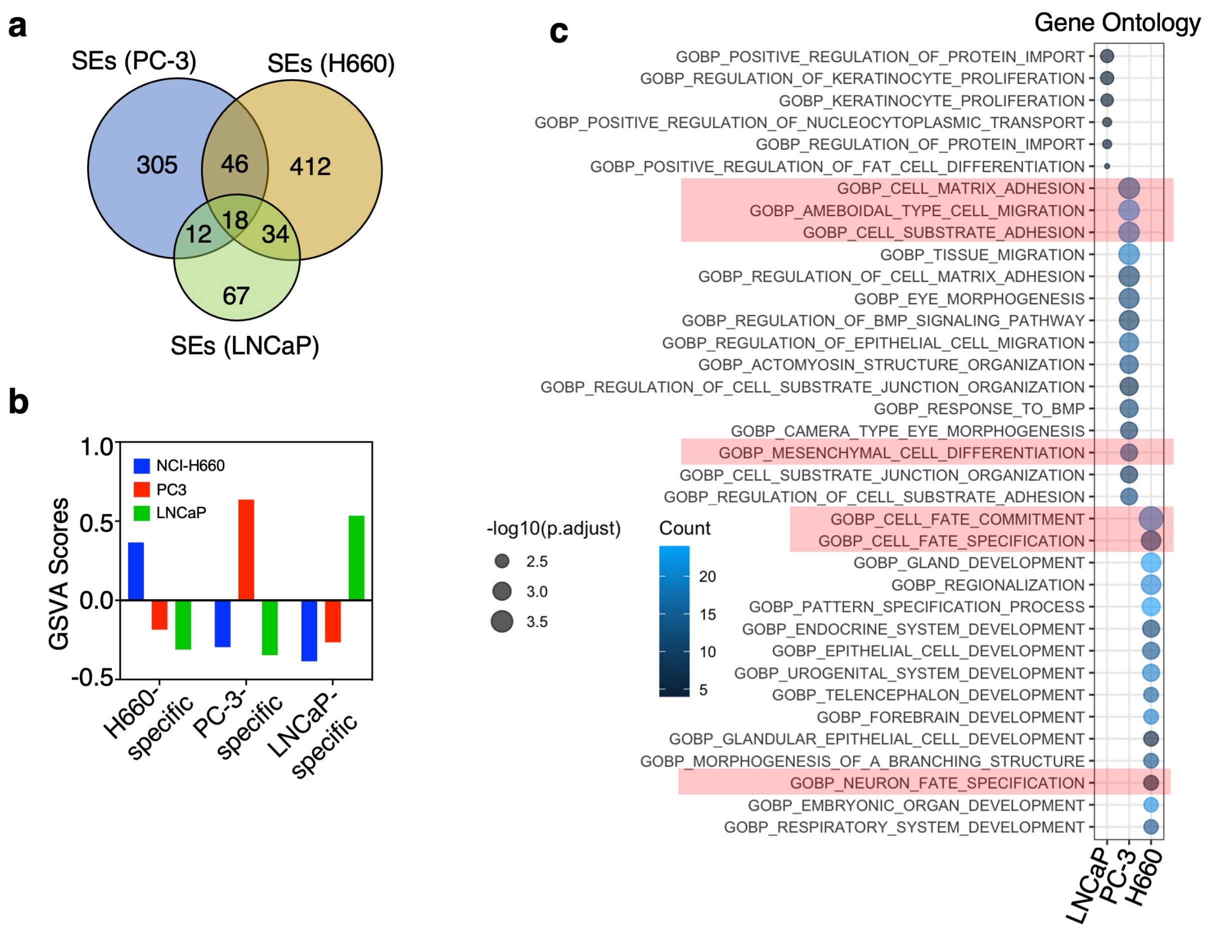


**Supplementary Fig. 10| Super-enhancer-associated genes are functionally distinct in CRPC subtypes**

**a,** Venn diagram for super-enhancer (SE)-associated genes in LNCaP, PC3, and H660 cells. **b,** Gene set variation analysis (GSVA) scores of the specific SE-associated genes in LNCaP, PC3, and H660 cells. **c,** Gene ontology annotation analysis to examine the functional enrichment of SE-associated genes identified in LNCaP, PC3, and H660 cells.

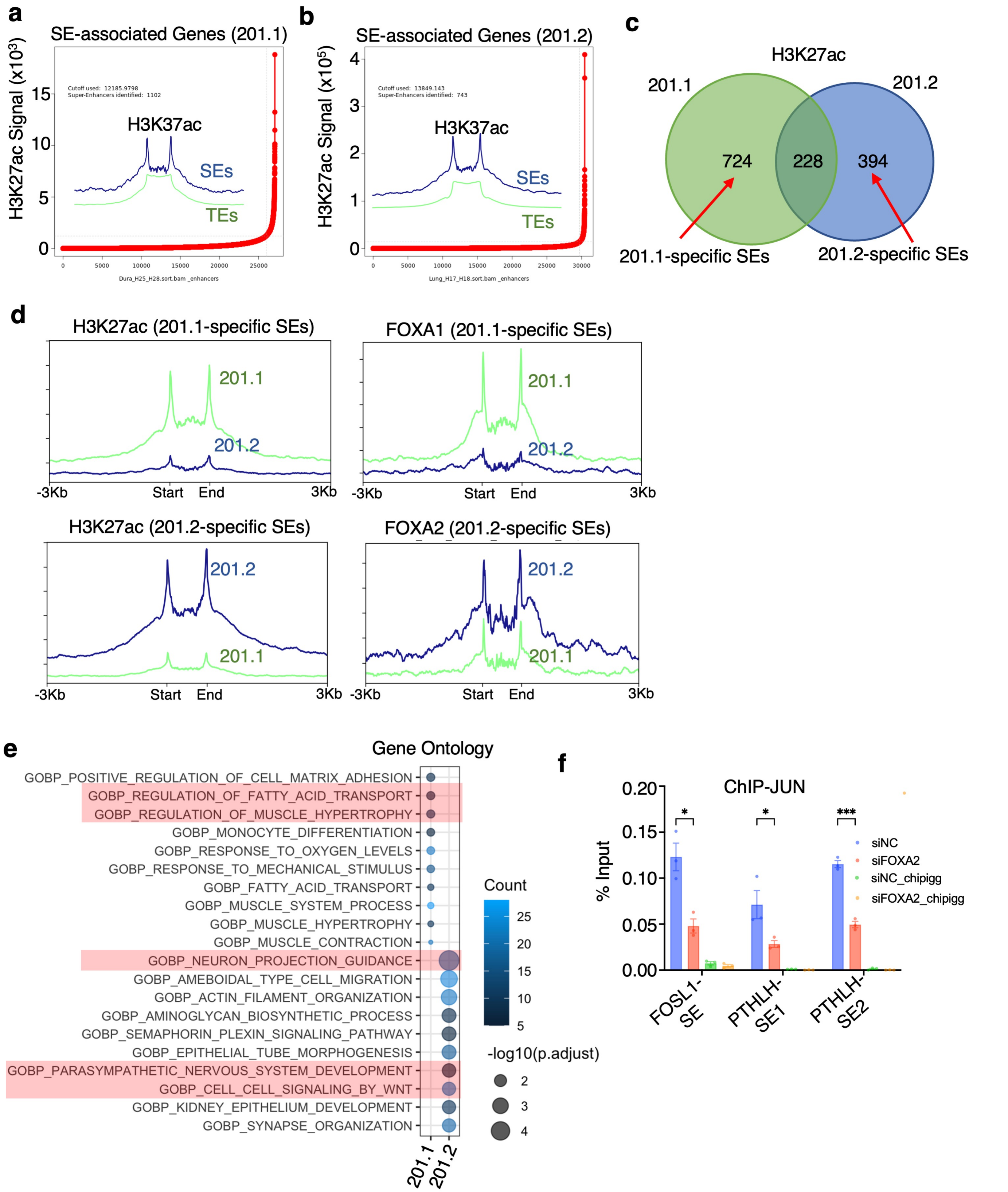


**Supplementary Fig. 11| Identification of super-enhancers in PDX models**

**a**, **b**, Model-specific super-enhancers (SEs) were identified using the ROSE algorithm based on ChIP-seq data

for H3K27ac in 201.1 (a) and 201.2 (b) models. **c,** Venn diagram for the overlap of SEs identified in 201.1 and 201.2 models. **d,** Average binding intensities of indicated proteins at 201.1-specific SEs and 201.2-specific SEs in 201.1 and 201.2 models. **e,** Gene ontology annotation analysis to examine the functional enrichment of differential SE-associated genes identified in 201.1 and 201.2 models. **f,** ChIP-qPCR for JUN binding at three SE sites in PC-3 cells transfected with siNTC or siFOXA2 (n=3 independent samples; data represented as mean ± SEM; statistical significance determined by unpaired two-sided *t*-test). ns ($P$>0.05), * (0.01<$P$<0.05), ** (0.001<$P$<0.01), *** ($P$<0.001), and **** ($P$<0.0001) were used to indicate the levels of *P*-value. Source data are provided as a Source Data file.

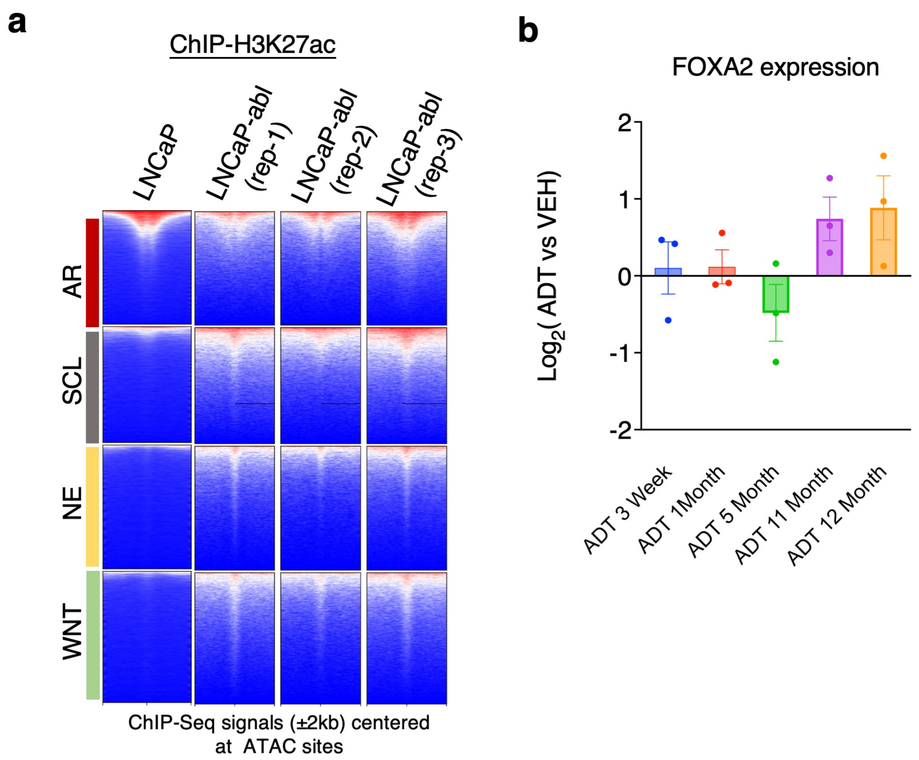


**Supplementary Fig. 12| AR targeting treatments drive tumor cells into a multilineage transition state**

**a**, Heatmap view for H3K27ac signal levels at four lineage-specific signature sites in LNCaP and LNCaP-abl cells (continuously cultured in hormone-depleted medium) using public ChIP-seq dataset (GSE114268 [3], GSE72467 [4]). **b**, FOXA2 expression in LNCaP cells cultured in hormone-depleted medium for indicated time points using public dataset (GSE8702 [5]) (Box plot - center: median; box: 25th to 75th IQR; whiskers: 1.5x IQR; outliers: individual data points, n=3 independent samples).

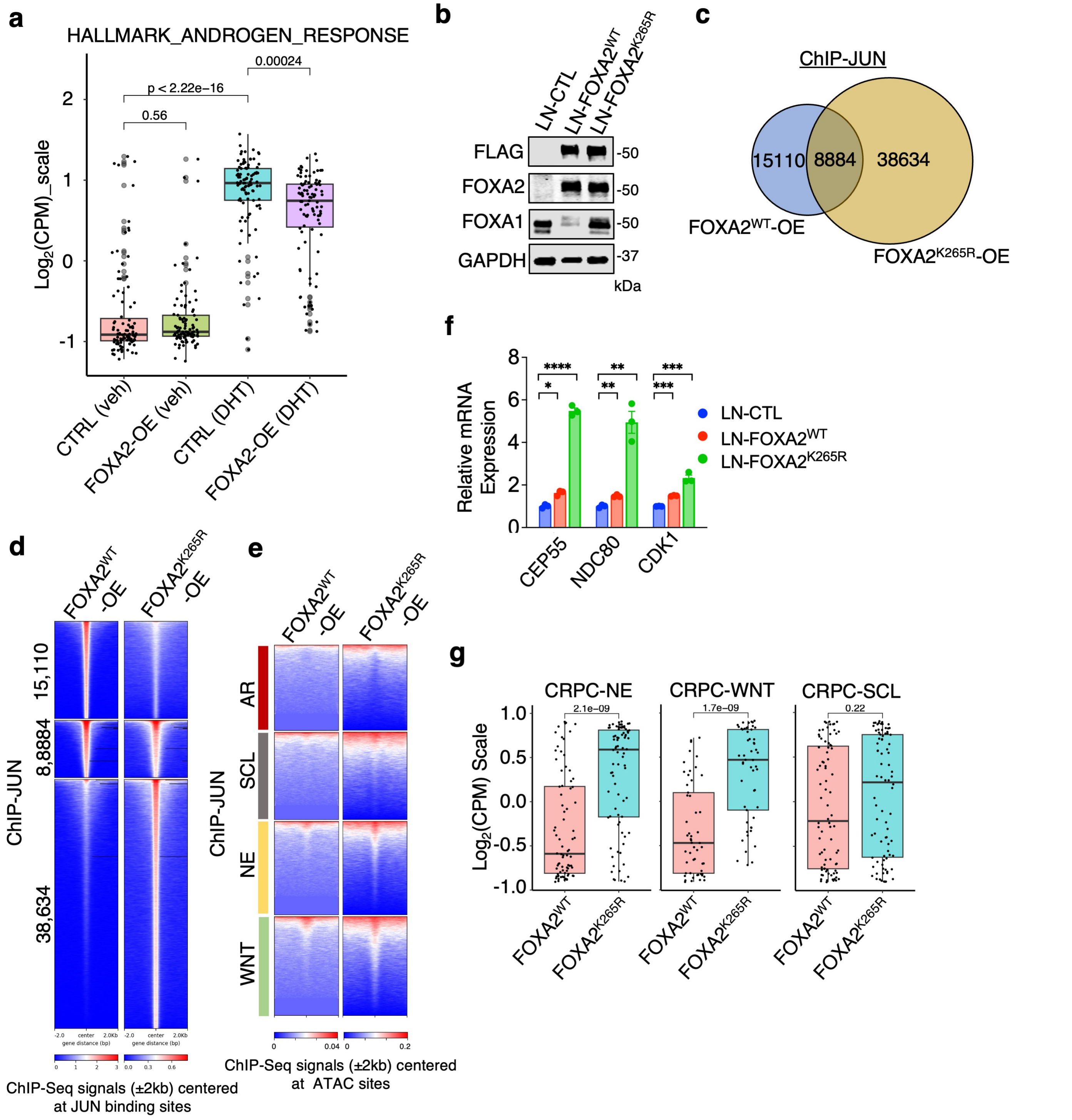


**Supplementary Fig. 13| K265R mutation of FOXA2 enhances JUN chromatin binding and maintains the multilineage state of tumor cells**

**a**, Boxplots (center: median; box: 25th to 75th IQR; whiskers: 1.5x IQR; outliers: individual data points; statistical significance determined by unpaired two-sided *t*-test) for expression levels of HALLMARK_ANDROGEN-

_RESPONSE gene set (n=100 genes) in control LNCaP cells or LN-FOXA2-OE cells treated with vehicle or 10nM DHT for 24 hours (based on RNA-seq data). **b**, Immunoblotting for indicated proteins in LNCaP cells overexpressing FLAG-tagged WT FOXA2 or K265R mutant (n=3 independent experiments). **c**, **d**, Venn diagram (c) or heatmap view (d) for JUN ChIP-seq peaks in the WT and K265R cell lines. **e**, Heatmap view for the ChIP-seq signal of JUN centered at specific chromatin sites exhibiting different ATAC signatures for CRPC subtypes [1]. **f**, qRT-PCR for indicated FOXA2/JUN targets in the WT and K265R cell lines (n=3 independent samples; data represented as mean ± SEM; statistical significance determined by unpaired two-sided *t*-test). **g**, Box plots (center: median; box: 25th to 75th IQR; whiskers: 1.5x IQR; outliers: individual data points; statistical significance determined by unpaired two-sided *t*-test) for three non-AR subtype-specific transcriptional signatures (n=93 genes for each subtype [1]) in the WT-expressing cells versus K265R mutant-expressing cells. ns ($P$>0.05), * (0.01<$P$<0.05), ** (0.001<$P$<0.01), *** ($P$<0.001), and **** ($P$<0.0001) were used to indicate the levels of $P$-value. Source data are provided as a Source Data file.

## OTHER SUPPLEMENTARY INFORMATION

### ChIP-qPCR Primers:

| Targets | Sequence (5'->3') |
|---|---|
| INCENP-F | TGTTGGCTAGAAGGCAAAGGAA |
| INCENP-R | CCAGTTTTCATCTGCTTTGGGT |
| CEP55-F | GAGAGCTGCCTGGCTTTTTA |
| CEP55-R | TGGCATCTTGTGTATCCATGTTTG |
| ERCC6L-F | CACACACCAGCCTGAGAGAAT |
| ERCC6L-R | GCTGGGTCTTAGGGAATGTGT |
| CCBE1_F | AGCTGTTTACTTTTTCATCCCGC |
| CCBE1_R | GGACCGGAGCTCCTTTTGTG |
| CDK1_62561458-F | AAAACAGCCTTCCAGGGAGTG |
| CDK1_62561458-R | ATCCAAGTCAAAGGTAGCTGGA |
| NDC80-F | GCCATGAGTCACAGAAGGTTG |
| NDC80-R | TCAGTGATAACCATACCAAACTGG |
| TP63_F | ACTCATCTGTTTACCTTTTGCTGT |
| TP63_R | TGTGGTTCTGAGGCTGAGTG |
| WNT7A_F | GCGTCAGTGAATGGTTGCTG |
| WNT7A_R | AAACTGGTTCCTGCCATCAG |
| PTHLH_F_SE1 | TGAGAGTTCAATGTTGCGAGTG |
| PTHLH_R_SE1 | TGATTTAACGTCAGGTGTGTGGT |
| PTHLH_F_SE2 | GGGTAGGGGAGGTGCATTTT |
| PTHLH_R_SE2 | TGTGTTGAGCCAGATACTATGTCA |
| FOSL1_F_SE | CAGGAGTGGGATGAAACGCC |
| FOSL1_R_SE | TGGGTGGGGTGGTTTATTGG |
| Mmp3_F | TTATTGACAGTGCAGACGGTCC |
| Mmp3_R | CTAGCCCAAGGCTTTTCAGGA |
| Mmp13_F | CCTTCGCCTCACTAGGAAGTT |
| Mmp13_R | CCCAGGGCAAGCATCTTCTAT |

### Mutagenesis Primers:

| | |
|---|---|
| **FOXA2_K265R** | |
| HmanFOXA2_K265R_Forward | CGCTTCAAGTGCGAGCGGCAGCTGGCGCTGAA |
| HumanFOXA2_K265R_Reverse | TTCAGCGCCAGCTGCCGCTCGCACTTGAAGCG |
| **FOXA2(1-165aa)** | |
| FOXA2_1-165aa_Forward | TAGAAGAGCCCGGGCGATCGCGAA |

| | |
|---|---|
| FOXA2_1-165aa_Reverse | CTTTGCGTGCGTGTAGCTGCGCCT |
| **FOXA2(1-324aa)** | |
| FOXA2_324aa_Forward | TAGAAGAGCCCGGGCGATCGCGAA |
| FOXA2_324aa_Reverse | GCCCCCTCGCTTGTGCTCCTGG |
| **FOXA2(165-463aa)** | |
| FOXA2_166-463aa_Forward | CCGCCCTACTCGTACATCTCGCTC |
| FOXA2_166-463aa_Reverse | CATGGTACCGAATTCCTTCAAGCCT |

**Designed siRNA Sequence:**

| | |
|---|---|
| siLSD1 | GACAAGCUGUUCCUAAAGAGAAA |

**Unprocessed Gel Blots:** The unprocessed immunoblotting gel blots for Supplementary Figures are provided in the Source Data file.

## SUPPLEMENTARY REFERENCES


1. Tang F, *et al.* Chromatin profiles classify castration-resistant prostate cancers suggesting therapeutic targets. *Science* **376**, eabe1505 (2022).

2. Abida W, *et al.* Genomic correlates of clinical outcome in advanced prostate cancer. *Proc Natl Acad Sci U S A* **116**, 11428-11436 (2019).

3. Gao S, *et al.* Chromatin binding of FOXA1 is promoted by LSD1-mediated demethylation in prostate cancer. *Nat Genet* **52**, 1011-1017 (2020).

4. Wang S, *et al.* Modeling cis-regulation with a compendium of genome-wide histone H3K27ac profiles. *Genome Res* **26**, 1417-1429 (2016).

5. D'Antonio JM, Ma C, Monzon FA, Pflug BR. Longitudinal analysis of androgen deprivation of prostate cancer cells identifies pathways to androgen independence. *Prostate* **68**, 698-714 (2008).
