# Supplementary material for: FOXA2 rewires AP-1 for transcriptional reprogramming and lineage plasticity in prostate cancer
Source: Nat Commun. 2024 Jun 8;15:4914. doi: 10.1038/s41467-024-49234-9 (PMC11162502; doi:10.1038/s41467-024-49234-9)
Supplement: Supplementary file 3 — Reporting Summary [file 41467_2024_49234_MOESM3_ESM.pdf]

nature portfolio

Corresponding author(s): Changmeng Cai

Last updated by author(s): May 20, 2024

Reporting Summary

Nature Portfolio wishes to improve the reproducibility of the work that we publish. This form provides structure for consistency and transparency in reporting. For further information on Nature Portfolio policies, see our Editorial Policies and the Editorial Policy Checklist.

Statistics

For all statistical analyses, confirm that the following items are present in the figure legend, table legend, main text, or Methods section.

n/a	Confirmed	
☐	☒	The exact sample size (*n*) for each experimental group/condition, given as a discrete number and unit of measurement
☐	☒	A statement on whether measurements were taken from distinct samples or whether the same sample was measured repeatedly
☐	☒	The statistical test(s) used AND whether they are one- or two-sided<br>*Only common tests should be described solely by name; describe more complex techniques in the Methods section.*
☐	☒	A description of all covariates tested
☒	☐	A description of any assumptions or corrections, such as tests of normality and adjustment for multiple comparisons
☐	☒	A full description of the statistical parameters including central tendency (e.g. means) or other basic estimates (e.g. regression coefficient) AND variation (e.g. standard deviation) or associated estimates of uncertainty (e.g. confidence intervals)
☐	☒	For null hypothesis testing, the test statistic (e.g. *F*, *t*, *r*) with confidence intervals, effect sizes, degrees of freedom and *P* value noted<br>*Give P values as exact values whenever suitable.*
☒	☐	For Bayesian analysis, information on the choice of priors and Markov chain Monte Carlo settings
☒	☐	For hierarchical and complex designs, identification of the appropriate level for tests and full reporting of outcomes
☒	☐	Estimates of effect sizes (e.g. Cohen's *d*, Pearson's *r*), indicating how they were calculated

Our web collection on statistics for biologists contains articles on many of the points above.

Software and code

Policy information about availability of computer code

Data collection	Raw Illumina output was converted to fastq format using Illumina Bcl2fastq
Data analysis	The ChIP-seq (and ATAC-seq) reads were mapped to the hg19 human genome using bwa (version 0.7.17-r1188) with the aln and sampe sub-commands. Samtools (version 1.9) was used to convert sam files to bam format. Enriched ChIP regions were evaluated using MACS2 (version 2.1.4) (49). The Intervene (version 0.6.5) was used to analyze peak intervals, determine overlapped regions, and generate Venn diagrams. The signals associated with genomic regions were visualized using compueMatrix and plotHeatmap tools from deepTools (version 3.3.0). computeMatrix was used to calculate scores for each genomic region and plotHeatmap was used to create a heatmap for scores associated with genomic regions. Motif enrichment analysis was performed using SeqPos with default settings. Binding and Expression Target Analysis (BETA) was performed using the BETA software package (version 1.0.7).<br>For RNA-seq The human reference genome (hg19) was used to align transcriptome-sequencing reads using STAR (version 2.7.1a). featureCounts (version 2.0.1) from GRCh37 Ensembl reference was used for counting. R package Edger (3.36.0) was then employed to process all gene counts and evaluate the differential expression using the Benjamini-Hochberg false discovery rate (FDR)-adjusted P-value. The expression values were normalized by centering and scaling across samples and displayed using the ComplexHeatmap (version 2.10.0) R package. Gene Set Enrichment Analysis (GSEA) was performed using Software GSEA (version 4.2.2) and R package fgsea (version 1.20.0).

For manuscripts utilizing custom algorithms or software that are central to the research but not yet described in published literature, software must be made available to editors and reviewers. We strongly encourage code deposition in a community repository (e.g. GitHub). See the Nature Portfolio guidelines for submitting code & software for further information.

Data

Policy information about availability of data

All manuscripts must include a data availability statement. This statement should provide the following information, where applicable:

- Accession codes, unique identifiers, or web links for publicly available datasets
- A description of any restrictions on data availability
- For clinical datasets or third party data, please ensure that the statement adheres to our policy

All the high-throughput sequencing data have been deposited in GEO under the accession number GSE232555

Research involving human participants, their data, or biological material

Policy information about studies with human participants or human data. See also policy information about sex, gender (identity/presentation), and sexual orientation and race, ethnicity and racism.

Reporting on sex and gender	Patients with prostate cancer are of male sex, so no patients of female sex were included.
Reporting on race, ethnicity, or other socially relevant groupings	N/A race, ethnicity and socially relevant groups of the patients who donated tissue were not collected for this study.
Population characteristics	N/A as this study used patient-derived xenografts (PDXs) from patient specimens that were obtained with informed consent
Recruitment	All human tissues were obtained with informed, written consent by an independent clinical coordinator
Ethics oversight	The studies were conducted under Human Research Ethics Committee (Institutional Review Board) approvals at Monash University (7996, 12287) and the Peter MacCallum Cancer Centre (15/98, 97_27).

Note that full information on the approval of the study protocol must also be provided in the manuscript.

Field-specific reporting

Please select the one below that is the best fit for your research. If you are not sure, read the appropriate sections before making your selection.

☒ Life sciences ☐ Behavioural & social sciences ☐ Ecological, evolutionary & environmental sciences

For a reference copy of the document with all sections, see nature.com/documents/nr-reporting-summary-flat.pdf

Life sciences study design

All studies must disclose on these points even when the disclosure is negative.

Sample size	Sample size was determined according to experimental design. No sample size calculation was necessary or performed. All biologic specimens available were used and included in the analysis
Data exclusions	No data were excluded.
Replication	Experiments were generally conducted with at lease three independent replicates, and these replicating experiments consistently yielded similar results. For ChIP-seq analyses in cell lines, two to three technical duplicates were performed and then merged for analysis. For ChIP-seq analyses in PDX samples, a minimum of two biological replicates were performed.
Randomization	Samples were randomly allocated
Blinding	The experiments were performed blinded

Reporting for specific materials, systems and methods

We require information from authors about some types of materials, experimental systems and methods used in many studies. Here, indicate whether each material, system or method listed is relevant to your study. If you are not sure if a list item applies to your research, read the appropriate section before selecting a response.

Materials & experimental systems

n/a	Involved in the study
☐	☒ Antibodies
☐	☒ Eukaryotic cell lines
☒	☐ Palaeontology and archaeology
☐	☒ Animals and other organisms
☒	☐ Clinical data
☒	☐ Dual use research of concern
☒	☐ Plants

Methods

n/a	Involved in the study
☐	☒ ChIP-seq
☒	☐ Flow cytometry
☒	☐ MRI-based neuroimaging

Antibodies

Antibodies used

anti-FOXA1 antibody (Abcam, ab23738, WB:1:2000), anti-FOXA2 antibody (Millipore, 17-10258, WB: 1:1000), anti-FOXA2 antibody (Abcam, ab256493, WB: 1:1000),anti-FOXA2 antibody (Abcam,ab108396), anti-H3K4me2 antibody (Milipore, 07-030, WB:1:1000), anti-H3K27ac antibody (Abcam, ab4729), anti-V5 (Thermo Fisher, R960-25, WB:1:1000), anti-AR (Millipore, 06-680, WB:1:1000), anti-Rabbit IgG (Millipore, 12-370), Mouse IgG (Millipore, 12-371), anti-Methyl-lysine (Abcam, ab23366, WB:1:200), anti-LSD1 (Abcam, ab17721, WB: 1:1000), anti-GAPDH (Abcam, Ab8245, WB:1:5000), anti-FLAG (Sigma, F3040, WB: 1:1000), anti-JUN (CST, 9165S, WB: 1:1000), anti-FOSL1 (CST, 5281S, WB: 1:1000), anti-FOSL2 (CST, 19967S, WB: 1:1000), anti-FOS (CST, 2250S, WB: 1:1000), anti-FOSB (CST, 2251T, WB: 1:1000), anti-Phospho-Aurora A /Aurora B/Aurora C(CST, 2914T, WB:1:1000), anti-rabbit (LI-COR, IRDye 800CW, WB: 1:3000), anti-mouse (LI-COR, IRDye 680RD, WB: 1:3000)

Validation

FOXA1 antibody: reference - PMID: 32868907; https://www.abcam.com/products/primary-antibodies/foxa1-antibody-ab23738.html
H3K27ac antibody: reference - PMID: 32868907; https://www.abcam.com/products/primary-antibodies/histone-h3-acetyl-k27-antibody-chip-grade-ab4729.html
H3K4me2 antibody: reference - PMID: 32868907; https://www.emdmillipore.com/US/en/product/Anti-dimethyl-Histone-H3-Lys4-Antibody,MM_NF-07-030
FOXA2 antibody: reference - PMID: 32868907; https://www.emdmillipore.com/US/en/product/ChIPAb-FOXA2-ChIP-Validated-Antibody-and-Primer-Set,MM_NF-17-10258?ReferrerURL=https%3A%2F%2Fwww.google.com%2F&bd=1
FOXA2 antibody: reference - PMID:PMID: 33009820 https://www.abcam.com/en-hk/products/primary-antibodies/foxa2-antibody-epr22919-71-chip-grade-ab256493
FOXA2 antibody: reference -PMID: 28938408; https://www.abcam.com/products/primary-antibodies/foxa2-antibody-epr4465-chip-grade-ab108396.html
JUN antibody: reference - PMID: 35617398; https://www.cellsignal.com/products/primary-antibodies/c-jun-60a8-rabbit-mab/9165
FOSL1 antibody: reference - PMID: 35617398; https://www.cellsignal.com/products/primary-antibodies/fra1-d80b4-rabbit-mab/5281
FOSL2 antibody: reference - PMID: 33219226; https://www.cellsignal.com/products/primary-antibodies/fra2-d2f1e-rabbit-mab/19967
FOS antibody: reference - PMID: 37821650; https://www.cellsignal.com/products/primary-antibodies/c-fos-9f6-rabbit-mab/2250
FOSB antibody: reference - PMID: 32923607; https://www.cellsignal.com/products/primary-antibodies/fosb-5g4-rabbit-mab/2251
AR antibody: reference - - PMID: 32868907; https://www.emdmillipore.com/US/en/product/Anti-Androgen-Receptor-Antibody,MM_NF-06-680
anti-Phospho-Aurora A /Aurora B/Aurora C antibody: reference - -PMID: 33953309; https://www.cellsignal.com/products/primary-antibodies/phospho-aurora-a-thr288-aurora-b-thr232-aurora-c-thr198-d13a11-xp-rabbit-mab/2914
Rabbit IgG Antibody:reference - - PMID: 32868907;https://www.emdmillipore.com/US/en/product/Normal-Rabbit-IgG,MM_NF-12-370
Mouse IgG Antibody:reference - - PMID: 32868907;https://www.emdmillipore.com/US/en/product/Normal-Mouse-IgG,MM_NF-12-371
Methylated-lysine Antibody:reference - - PMID: 32868907;https://www.abcam.com/en-no/products/primary-antibodies/methylated-lysine-di-methyl-mono-methyl-antibody-ab23366
LSD1 Antibody:reference - - PMID: 31428587;https://www.abcam.com/products/primary-antibodies/kdm1lsd1-antibody-nuclear-marker-ab17721.html
GAPDH Antibody:reference - - PMID: 37549269;https://www.abcam.com/products/primary-antibodies/gapdh-antibody-6c5-loading-control-ab8245.html
FLAG Antibody:reference - - PMID: 37663929;https://www.sigmaaldrich.com/US/en/product/sigma/f3040
anti-rabbit: reference - PMID: 32868907; https://www.licor.com/bio/reagents/irdye-800cw-infrared-dyes
anti-mouse: reference - PMID: 32868907; https://www.licor.com/bio/reagents/irdye-680rd-infrared-dyes

Eukaryotic cell lines

Policy information about cell lines and Sex and Gender in Research

Cell line source(s)

PC-3, LNCaP, NCI-H660 and CWR-22Rv1 were purchased from ATCC. SKO and DKO cells were from Dr. Leigh Ellis' lab.

Authentication

Authenticated using short tandem repeat (STR) profiling

Mycoplasma contamination	Mycoplasma contamination was assessed using the MycoAlert kit from Lonza, and no contamination was detected.
Commonly misidentified lines (See ICLAC register)	No commonly misidentified cell lines were used

Animals and other research organisms

Policy information about studies involving animals; ARRIVE guidelines recommended for reporting animal research, and Sex and Gender in Research

Laboratory animals	Mouse, ICR SCID, male, 6 weeks; Zebrafish embryos, AB or Tubingen wild-type lines, male/female, 3 days post-fertilization
Wild animals	No wild animals were used in the study
Reporting on sex	The finding only applies to male since prostate cancer is a male-specific disease
Field-collected samples	No field collected samples were used in the study
Ethics oversight	Animal experiments were conducted in accordance with institutional and USA national guidelines and were approved by the Institutional Animal Care and Use Committee (IACUC) of University of Massachusetts Boston. Animal group sizes were estimated based on the power analysis using preliminary data and were approved by IACUC.

Note that full information on the approval of the study protocol must also be provided in the manuscript.

Plants

Seed stocks	N/A
Novel plant genotypes	N/A
Authentication	N/A

ChIP-seq

Data deposition

- [x] Confirm that both raw and final processed data have been deposited in a public database such as GEO.
- [x] Confirm that you have deposited or provided access to graph files (e.g. BED files) for the called peaks.

Data access links
May remain private before publication.

https://www.ncbi.nlm.nih.gov/geo/query/acc.cgi?acc=GSE232555

https://www.ncbi.nlm.nih.gov/geo/query/acc.cgi?acc=GSE114268
https://www.ncbi.nlm.nih.gov/geo/query/acc.cgi?acc=GSE72467
https://www.ncbi.nlm.nih.gov/geo/query/acc.cgi?acc=GSE8702
https://www.ncbi.nlm.nih.gov/geo/query/acc.cgi?acc=GSE137209
https://www.ncbi.nlm.nih.gov/geo/query/acc.cgi?acc=GSE52201
https://www.ncbi.nlm.nih.gov/geo/query/acc.cgi?token=mncvaaievfirruv&acc=GSE77448

Files in database submission

DKO_NTC_ChIPFOXA2_1_S1_L001_R1_001.fastq.gz
DKO_NTC_ChIPFOXA2_1_S1_L001_R2_001.fastq.gz
DKO_NTC_ChIPFOXA2_2_S2_L001_R1_001.fastq.gz
DKO_NTC_ChIPFOXA2_2_S2_L001_R2_001.fastq.gz
DKO_ORY10uM_ChIPFOXA2_1_S3_L001_R1_001.fastq.gz
DKO_ORY10uM_ChIPFOXA2_1_S3_L001_R2_001.fastq.gz
DKO_ORY10uM_ChIPFOXA2_2_S4_L001_R1_001.fastq.gz
DKO_ORY10uM_ChIPFOXA2_2_S4_L001_R2_001.fastq.gz
LN_FOXA2K265R_ChIPJUN_1_S3_L001_R1_001.fastq.gz
LN_FOXA2K265R_ChIPJUN_1_S3_L001_R2_001.fastq.gz
LN_FOXA2K265R_ChIPJUN_2_S4_L001_R1_001.fastq.gz
LN_FOXA2K265R_ChIPJUN_2_S4_L001_R2_001.fastq.gz
ab108396_PC3_Rep1_21-01620_R1.fastq.gz
ab256493_PC3_Rep1_21-01622_R1.fastq.gz
H660_ORY_ChIP-FOXA2_1_S23_L001_R1_001.fastq.gz
H660_ORY_ChIP-FOXA2_1_S23_L001_R2_001.fastq.gz
H660_ORY_ChIP-FOXA2_2_S24_L001_R1_001.fastq.gz
H660_ORY_ChIP-FOXA2_2_S24_L001_R2_001.fastq.gz
H660_Veh_ChIP-FOXA2_1_S21_L001_R1_001.fastq.gz
H660_Veh_ChIP-FOXA2_1_S21_L001_R2_001.fastq.gz

H660_Veh_ChIP-FOXA2_2_S22_L001_R1_001.fastq.gz
H660_Veh_ChIP-FOXA2_2_S22_L001_R2_001.fastq.gz
H660_Veh_ChIP_H3K4me2_1_S23_L001_R1_001.fastq.gz
H660_Veh_ChIP_H3K4me2_1_S23_L001_R2_001.fastq.gz
H660_Veh_ChIP_H3K4me2_2_S24_L001_R1_001.fastq.gz
H660_Veh_ChIP_H3K4me2_2_S24_L001_R2_001.fastq.gz
H660_siFOXA2_ChIP-JUN_1_S19_L001_R1_001.fastq.gz
H660_siFOXA2_ChIP-JUN_1_S19_L001_R2_001.fastq.gz
H660_siFOXA2_ChIP-JUN_2_S20_L001_R1_001.fastq.gz
H660_siFOXA2_ChIP-JUN_2_S20_L001_R2_001.fastq.gz
H660_siNC_ChIP-JUN_1_S17_L001_R1_001.fastq.gz
H660_siNC_ChIP-JUN_1_S17_L001_R2_001.fastq.gz
H660_siNC_ChIP-JUN_2_S18_L001_R1_001.fastq.gz
H660_siNC_ChIP-JUN_2_S18_L001_R2_001.fastq.gz
H660_siNTC_VEH_ATAC_S1_L001_R1_001.fastq.gz
H660_siNTC_VEH_ATAC_S1_L001_R2_001.fastq.gz
LN_FOXA2OE_ChIPFOXA2_1_S1_L001_R1_001.fastq.gz
LN_FOXA2OE_ChIPFOXA2_1_S1_L001_R2_001.fastq.gz
LN_FOXA2OE_ChIPFOXA2_2_S2_L001_R1_001.fastq.gz
LN_FOXA2OE_ChIPFOXA2_2_S2_L001_R2_001.fastq.gz
LN_FOXA2OE_ChIP_JUN_1_S5_L001_R1_001.fastq.gz
LN_FOXA2OE_ChIP_JUN_1_S5_L001_R2_001.fastq.gz
LN_FOXA2OE_ChIP_JUN_2_S6_L001_R1_001.fastq.gz
LN_FOXA2OE_ChIP_JUN_2_S6_L001_R2_001.fastq.gz
LN_siFOXA1_ChIP_JUN_1_S15_L001_R1_001.fastq.gz
LN_siFOXA1_ChIP_JUN_1_S15_L001_R2_001.fastq.gz
LN_siFOXA1_ChIP_JUN_2_S16_L001_R1_001.fastq.gz
LN_siFOXA1_ChIP_JUN_2_S16_L001_R2_001.fastq.gz
LN_siNC_ChIP_JUN_1_S13_L001_R1_001.fastq.gz
LN_siNC_ChIP_JUN_1_S13_L001_R2_001.fastq.gz
LN_siNC_ChIP_JUN_2_S14_L001_R1_001.fastq.gz
LN_siNC_ChIP_JUN_2_S14_L001_R2_001.fastq.gz
PC3_C12_CHIP-FOXA2_1_S5_L001_R1_001.fastq.gz
PC3_C12_CHIP-FOXA2_1_S5_L001_R2_001.fastq.gz
PC3_C12_CHIP-FOXA2_2_S6_L001_R1_001.fastq.gz
PC3_C12_CHIP-FOXA2_2_S6_L001_R2_001.fastq.gz
PC3_ORY_CHIP-FOXA2_1_S1_L001_R1_001.fastq.gz
PC3_ORY_CHIP-FOXA2_1_S1_L001_R2_001.fastq.gz
PC3_ORY_CHIP-FOXA2_2_S2_L001_R1_001.fastq.gz
PC3_ORY_CHIP-FOXA2_2_S2_L001_R2_001.fastq.gz
PC3_VEH_CHIP-FOXA2_1_S3_L001_R1_001.fastq.gz
PC3_VEH_CHIP-FOXA2_1_S3_L001_R2_001.fastq.gz
PC3_VEH_CHIP-FOXA2_2_S4_L001_R1_001.fastq.gz
PC3_VEH_CHIP-FOXA2_2_S4_L001_R2_001.fastq.gz
PC3_siFOXA2_ChIP_FOSL1_1_S3_L001_R1_001.fastq.gz
PC3_siFOXA2_ChIP_FOSL1_1_S3_L001_R2_001.fastq.gz
PC3_siFOXA2_ChIP_FOSL1_2_S4_L001_R1_001.fastq.gz
PC3_siFOXA2_ChIP_FOSL1_2_S4_L001_R2_001.fastq.gz
PC3_siFOXA2_ChIP_JUN_1_S15_L001_R1_001.fastq.gz
PC3_siFOXA2_ChIP_JUN_1_S15_L001_R2_001.fastq.gz
PC3_siFOXA2_ChIP_JUN_2_S16_L001_R1_001.fastq.gz
PC3_siFOXA2_ChIP_JUN_2_S16_L001_R2_001.fastq.gz
PC3_siNC_ChIP_FOSL1_1_S1_L001_R1_001.fastq.gz
PC3_siNC_ChIP_FOSL1_1_S1_L001_R2_001.fastq.gz
PC3_siNC_ChIP_FOSL1_2_S2_L001_R1_001.fastq.gz
PC3_siNC_ChIP_FOSL1_2_S2_L001_R2_001.fastq.gz
PC3_siNC_ChIP_JUN_1_S13_L001_R1_001.fastq.gz
PC3_siNC_ChIP_JUN_1_S13_L001_R2_001.fastq.gz
PC3_siNC_ChIP_JUN_2_S14_L001_R1_001.fastq.gz
PC3_siNC_ChIP_JUN_2_S14_L001_R2_001.fastq.gz
PC3_siNTC_VEH_ATAC_S4_L001_R1_001.fastq.gz
PC3_siNTC_VEH_ATAC_S4_L001_R2_001.fastq.gz
PC3_siNC_ChIP_H3K27AC_1_S1_L001_R1_001.fastq.gz
PC3_siNC_ChIP_H3K27AC_1_S1_L001_R2_001.fastq.gz
PC3_siNC_ChIP_H3K27AC_2_S2_L001_R1_001.fastq.gz
PC3_siNC_ChIP_H3K27AC_2_S2_L001_R2_001.fastq.gz
DKO_NTC_ChIPFOXA2_treat.bw
DKO_ORY10uM_ChIPFOXA2_treat.bw

LN_FOXA2K265R_ChIPJUN_treat_pileup.bw
ab108396_PC3_Rep1_21-01620_R1_treat_pileup.bw
ab256493_PC3_Rep1_21-01622_R1_treat_pileup.bw
H660_ORY_ChIP_FOXA2_treat_pileup.bw
H660_Veh_ChIP_FOXA2_treat_pileup.bw
H660_Veh_ChIP_H3K4me2.bw
H660_siFOXA2_ChIP_JUN_treat_pileup.bw
H660_siNC_ChIP_JUN_treat_pileup.bw
H660_siNTC_VEH_ATAC_treat.bw
LN_FOXA2OE_ChIPFOXA2_treat.bw
LN_FOXA2OE_ChIP_JUN_treat.bw
LN_siFOXA1_ChIP_JUN_treat_pileup.bw
LN_siNC_ChIP_JUN_treat_pileup.bw
PC3_C12_CHIP-FOXA2_treat_pileup.bw
PC3_ORY_CHIP-FOXA2_treat_pileup.bw
PC3_VEH_CHIP-FOXA2_treat_pileup.bw
PC3_siFOXA2_ChIP_FOSL1_treat.bw
PC3_siFOXA2_ChIP_JUN.bw
PC3_siNC_ChIP_FOSL1_treat.bw
PC3_siNC_ChIP_JUN.bw
PC3_siNTC_VEH_ATAC_treat.bw
DKO_NTC_ChIPFOXA2_peaks.bed
DKO_ORY10uM_ChIPFOXA2_peaks.bed
LN_FOXA2K265R_ChIPJUN_peaks.bed
ab108396_PC3_Rep1_21-01620_R1_peaks.bed
ab256493_PC3_Rep1_21-01622_R1_peaks.bed
H660_ORY_ChIP_FOXA2.bed
H660_Veh_ChIP_FOXA2.bed
H660_Veh_ChIP_H3K4me2.bed
H660_siFOXA2_ChIP_JUN.bed
H660_siNC_ChIP_JUN.bed
H660_siNTC_VEH_ATAC_peaks.bed
LN_FOXA2OE_ChIPFOXA2_peaks.bed
LN_FOXA2OE_ChIP_JUN_peaks.bed
LN_siFOXA1_ChIP_JUN.bed
LN_siNC_ChIP_JUN.bed
PC3_C12_CHIP-FOXA2_peaks.bed
PC3_ORY_CHIP-FOXA2_peaks.bed
PC3_VEH_CHIP-FOXA2_peaks.bed
PC3_siFOXA2_ChIP_FOSL1.bed
PC3_siFOXA2_ChIP_JUN_peaks.bed
PC3_siNC_ChIP_FOSL1.bed
PC3_siNC_ChIP_JUN_peaks.bed
PC3_siNTC_VEH_ATAC_peaks.bed
PC3_siNC_ChIP_H3K27AC.bw
PC3_siNC_ChIP_H3K27AC_peaks.bed
201.1_201_2_PDX_Pooled_Input.bigwig
201.1_PDX_AR_Rep1(H7).bigwig
201.1_PDX_AR_Rep2(H9).bigwig
201.1_PDX_FOXA1_Rep1(D25).bigwig
201.1_PDX_FOXA1_Rep2(D26).bigwig
201.1_PDX_FOXA1_Rep3(D27).bigwig
201.1_PDX_FOXA1_Rep3(D28).bigwig
201.1_PDX_H3K27ac_Rep1(D25).bigwig
201.1_PDX_H3K27ac_Rep1(H9).bigwig
201.1_PDX_H3K27ac_Rep2(D28).bigwig
201.2_PDX_FOXA2_ab108396_Rep1(L17).bigwig
201.2_PDX_FOXA2_ab108396_Rep2(L18).bigwig
201.2_PDX_FOXA2_ab256493_Rep1(L17).bigwig
201.2_PDX_FOXA2_ab256493_Rep2(L18).bigwig
201.2_PDX_H3K27ac_Rep1(L17).bigwig
201.2_PDX_H3K27ac_Rep2(L18).bigwig

Genome browser session (e.g. UCSC)

N/A

Methodology

Replicates

Individual cell line ChIP-seq, technical duplicates were performed and then merged for analysis. Regarding individual PDX samples'

ChIP-seq, they were profiled with a minimum of two biological replicates

Sequencing depth

ChIP-seq were 51bp paired-end
sample_name Unique Reads Duplicate Reads
DKO_NTC_ChIPFOXA2_1_S1_L001_R1_001 7137911 23843626
DKO_NTC_ChIPFOXA2_1_S1_L001_R2_001 7514199 23467338
DKO_NTC_ChIPFOXA2_2_S2_L001_R1_001 6376438 20688062
DKO_NTC_ChIPFOXA2_2_S2_L001_R2_001 6706406 20358094
DKO_ORY10uM_ChIPFOXA2_1_S3_L001_R1_001 11533740 26390444
DKO_ORY10uM_ChIPFOXA2_1_S3_L001_R2_001 12038918 25885266
DKO_ORY10uM_ChIPFOXA2_2_S4_L001_R1_001 11656330 22933132
DKO_ORY10uM_ChIPFOXA2_2_S4_L001_R2_001 12062048 22527414
LN_FOXA2K265R_ChIPFOXA2_1_S1_L001_R1_001 11204891 17305805
LN_FOXA2K265R_ChIPFOXA2_1_S1_L001_R2_001 11569023 16941673
LN_FOXA2K265R_ChIPFOXA2_2_S2_L001_R1_001 10852509 13895563
LN_FOXA2K265R_ChIPFOXA2_2_S2_L001_R2_001 11154851 13593221
ab108396_PC3_Rep1_21-01620_R1 22597891 1857777
ab256493_PC3_Rep1_21-01622_R1 23993484 1690731
H660_ORY_ChIP_FOXA2_1_S23_L001_R1_001 13398797 18914161
H660_ORY_ChIP_FOXA2_1_S23_L001_R2_001 13515239 18914161
H660_ORY_ChIP_FOXA2_2_S24_L001_R1_001 13351508 19103741
H660_ORY_ChIP_FOXA2_2_S24_L001_R2_001 13464894 19103741
H660_Veh_ChIP_FOXA2_1_S21_L001_R1_001 12919541 19072842
H660_Veh_ChIP_FOXA2_1_S21_L001_R2_001 13021927 19072842
H660_Veh_ChIP_FOXA2_2_S22_L001_R1_001 5599247 7265942
H660_Veh_ChIP_FOXA2_2_S22_L001_R2_001 5623740 7265942
H660_Veh_ChIP_H3K4me2_1_S23_L001_R1_001 10520329 11789280
H660_Veh_ChIP_H3K4me2_1_S23_L001_R2_001 10506242 11789280
H660_Veh_ChIP_H3K4me2_2_S24_L001_R1_001 9364057 10472299
H660_Veh_ChIP_H3K4me2_2_S24_L001_R2_001 9351644 10472299
H660_siFOXA2_ChIP-JUN_1_S19_L001_R1_001 13942243 19120687
H660_siFOXA2_ChIP-JUN_1_S19_L001_R2_001 14062194 19120687
H660_siFOXA2_ChIP-JUN_2_S20_L001_R1_001 15644963 22248440
H660_siFOXA2_ChIP-JUN_2_S20_L001_R2_001 15799960 22248440
H660_siNC_ChIP-JUN_1_S17_L001_R1_001 12874805 17330477
H660_siNC_ChIP-JUN_1_S17_L001_R2_001 12961817 17330477
H660_siNC_ChIP-JUN_2_S18_L001_R1_001 14259534 19464930
H660_siNC_ChIP-JUN_2_S18_L001_R2_001 14345142 19464930
H660_siNTC_VEH_ATAC_S1_L001_R1_001 41057790 74506192
H660_siNTC_VEH_ATAC_S1_L001_R2_001 41233498 74506192
LN_FOXA2OE_ChIPFOXA2_1_S1_L001_R1_001 50604697 76129836
LN_FOXA2OE_ChIPFOXA2_1_S1_L001_R2_001 51875569 76129836
LN_FOXA2OE_ChIPFOXA2_2_S2_L001_R1_001 38590985 53069065
LN_FOXA2OE_ChIPFOXA2_2_S2_L001_R2_001 39322064 53069065
LN_FOXA2OE_ChIP_JUN_1_S5_L001_R1_001 8489204 22019857
LN_FOXA2OE_ChIP_JUN_1_S5_L001_R2_001 8688696 22019857
LN_FOXA2OE_ChIP_JUN_2_S6_L001_R1_001 9291303 27006477
LN_FOXA2OE_ChIP_JUN_2_S6_L001_R2_001 9506232 27006477
LN_siFOXA1_ChIP_JUN_1_S15_L001_R1_001 12138693 22516428
LN_siFOXA1_ChIP_JUN_1_S15_L001_R2_001 12327027 22516428
LN_siFOXA1_ChIP_JUN_2_S16_L001_R1_001 13269793 25398565
LN_siFOXA1_ChIP_JUN_2_S16_L001_R2_001 13485426 25398565
LN_siNC_ChIP_JUN_1_S13_L001_R1_001 11139988 18642165
LN_siNC_ChIP_JUN_1_S13_L001_R2_001 11273004 18642165
LN_siNC_ChIP_JUN_2_S14_L001_R1_001 11010900 17245893
LN_siNC_ChIP_JUN_2_S14_L001_R2_001 11116779 17245893
PC3_C12_CHIP-FOXA2_1_S5_L001_R1_001 12448066 16732083
PC3_C12_CHIP-FOXA2_1_S5_L001_R2_001 12526898 16732083
PC3_C12_CHIP-FOXA2_2_S6_L001_R1_001 12955538 17375112
PC3_C12_CHIP-FOXA2_2_S6_L001_R2_001 13029917 17375112
PC3_ORY_CHIP-FOXA2_1_S1_L001_R1_001 15425770 23852117
PC3_ORY_CHIP-FOXA2_1_S1_L001_R2_001 15598523 23852117
PC3_ORY_CHIP-FOXA2_2_S2_L001_R1_001 12558159 18536898
PC3_ORY_CHIP-FOXA2_2_S2_L001_R2_001 12693449 18536898
PC3_VEH_CHIP-FOXA2_1_S3_L001_R1_001 16176211 24798100
PC3_VEH_CHIP-FOXA2_1_S3_L001_R2_001 16352871 24798100
PC3_VEH_CHIP-FOXA2_2_S4_L001_R1_001 13985473 21400151
PC3_VEH_CHIP-FOXA2_2_S4_L001_R2_001 14130647 21400151
PC3_siFOXA2_ChIP_FOSL1_1_S3_L001_R1_001 10675306 23734940

PC3_siFOXA2_ChIP_FOSL1_1_S3_L001_R2_001 10909423 23734940
PC3_siFOXA2_ChIP_FOSL1_2_S4_L001_R1_001 10810477 22575968
PC3_siFOXA2_ChIP_FOSL1_2_S4_L001_R2_001 11036338 22575968
PC3_siFOXA2_ChIP_JUN_1_S15_L001_R1_001 10960598 14209286
PC3_siFOXA2_ChIP_JUN_1_S15_L001_R2_001 10975196 14209286
PC3_siFOXA2_ChIP_JUN_2_S16_L001_R1_001 8140874 10501364
PC3_siFOXA2_ChIP_JUN_2_S16_L001_R2_001 8155286 10501364
PC3_siNC_ChIP_FOSL1_1_S1_L001_R1_001 14327435 22122565
PC3_siNC_ChIP_FOSL1_1_S1_L001_R2_001 14490936 22122565
PC3_siNC_ChIP_FOSL1_2_S2_L001_R1_001 14910761 22911154
PC3_siNC_ChIP_FOSL1_2_S2_L001_R2_001 15090202 22911154
PC3_siNC_ChIP_JUN_1_S13_L001_R1_001 13307903 18167930
PC3_siNC_ChIP_JUN_1_S13_L001_R2_001 13345814 18167930
PC3_siNC_ChIP_JUN_2_S14_L001_R1_001 13241267 17866678
PC3_siNC_ChIP_JUN_2_S14_L001_R2_001 13288283 17866678
PC3_siNTC_VEH_ATAC_S4_L001_R1_001 72912270 115177815
PC3_siNTC_VEH_ATAC_S4_L001_R2_001 73007758 115177815
PC3_siNC_ChIP_H3K27AC_1_S1_L001_R1_001 24234276 28496885
PC3_siNC_ChIP_H3K27AC_1_S1_L001_R2_001 24215934 28496885
PC3_siNC_ChIP_H3K27AC_2_S2_L001_R1_001 29432323 35308949
PC3_siNC_ChIP_H3K27AC_2_S2_L001_R2_001 29459460 35308949

Antibodies

anti-FOXA2 antibody (Millipore, 17-10258), anti-FOXA2 antibody (Abcam,ab108396), anti-FOXA2 antibody (Abcam,ab256493), anti-H3K4me2 antibody (Milipore, 07-030), anti-H3K27ac antibody (Abcam, ab4729), anti-JUN (CST, 9165S), anti-FOSL1 (CST, 5281S)

Peak calling parameters

macs2 callpeak -g hs --bw 250 --mfold 10 30 -f BAMPE --extsize 100 --seed 1 --fix-bimodal --qvalue 0.05 --SPMR -B

Data quality

ChIP-seq data were demonstrated to be of high quality through various assessments. Fastqc was executed on all samples to confirm the presence of good quality data. Additionally, the number of peaks was considered, with a cutoff of FDR <= 0.05.
Sample_name Peak_number
DKO_NTC_ChIPFOXA2_peaks 5715
DKO_ORY10uM_ChIPFOXA2_peaks 44943
LN_FOXA2K265R_ChIPJUN_peaks 47505
ab108396_PC3_Rep1_21-01620_R1_peaks 77822
ab256493_PC3_Rep1_21-01622_R1_peaks 62714
H660_ORY_ChIP_FOXA2 11469
H660_Veh_ChIP_FOXA2 11886
H660_siFOXA2_ChIP_JUN 59452
H660_siNC_ChIP_JUN 18401
H660_siNTC_VEH_ATAC_peaks 99959
H660_Veh_ChIP_H3K4me2 70388
LN_FOXA2OE_ChIP_JUN_peaks 23994
LN_FOXA2OE_ChIPFOXA2_peaks 2500
LN_siFOXA1_ChIP_JUN 1964
LN_siNC_ChIP_JUN 2421
PC3_VEH_CHIP-FOXA2_peaks 24038
PC3_C12_CHIP-FOXA2_peaks 8352
PC3_ORY_CHIP-FOXA2_peaks 13732
PC3_siFOXA2_ChIP_FOSL1 1538
PC3_siNC_ChIP_FOSL1 3843
PC3_siFOXA2_ChIP_JUN_peaks 4081
PC3_siNC_ChIP_JUN_peaks 23890
PC3_siNC_ChIP_H3K27AC_peaks 90468
PC3_siNTC_VEH_ATAC_peaks 210960

Software

MACS2 (version 2.1.4)
